# Understanding species limits through the formation of phylogeographic lineages

**DOI:** 10.1002/ece3.70263

**Published:** 2024-10-02

**Authors:** Frank T. Burbrink, Edward A. Myers, R. Alexander Pyron

**Affiliations:** ^1^ Department of Herpetology American Museum of Natural History New York New York USA; ^2^ Department of Herpetology California Academy of Sciences San Francisco California USA; ^3^ Department of Biological Sciences The George Washington University Washington DC USA

**Keywords:** genome cline, genome selection, hybrid zone, spatial cline, speciation

## Abstract

The outcomes of speciation across organismal dimensions (e.g., ecological, genetic, phenotypic) are often assessed using phylogeographic methods. At one extreme, reproductively isolated lineages represent easily delimitable species differing in many or all dimensions, and at the other, geographically distinct genetic segments introgress across broad environmental gradients with limited phenotypic disparity. In the ambiguous gray zone of speciation, where lineages are genetically delimitable but still interacting ecologically, it is expected that these lineages represent species in the context of ontology and the evolutionary species concept when they are maintained over time with geographically well‐defined hybrid zones, particularly at the intersection of distinct environments. As a result, genetic structure is correlated with environmental differences and not space alone, and a subset of genes fail to introgress across these zones as underlying genomic differences accumulate. We present a set of tests that synthesize species delimitation with the speciation process. We can thereby assess historical demographics and diversification processes while understanding how lineages are maintained through space and time by exploring spatial and genome clines, genotype‐environment interactions, and genome scans for selected loci. Employing these tests in eight lineage‐pairs of snakes in North America, we show that six pairs represent 12 “good” species and that two pairs represent local adaptation and regional population structure. The distinct species pairs all have the signature of divergence before or near the mid‐Pleistocene, often with low migration, stable hybrid zones of varying size, and a subset of loci showing selection on alleles at the hybrid zone corresponding to transitions between distinct ecoregions. Locally adapted populations are younger, exhibit higher migration, and less ecological differentiation. Our results demonstrate that interacting lineages can be delimited using phylogeographic and population genetic methods that properly integrate spatial, temporal, and environmental data.

## Graphical abstract

We present a set of tests to examine processes of speciation that will help delimit taxa given deep phylogeographic history and interaction within hybrid zones. Outcomes from distinct phylogeographic pairs of snakes in North America all have the signature of divergence before or near the mid‐Pleistocene often with low migration. For these pairs we demonstrate the presence of stable hybrid zones of varying size with a subset of loci showing selection on alleles at the hybrid zone corresponding to transitions between very distinct ecoregions.

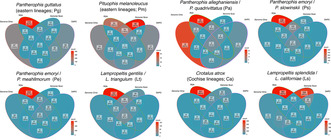

## 1 INTRODUCTION

Mechanisms of speciation across biomes and their contribution to biodiversity at the continental scale remain poorly known. Exploring diversification using phylogenetic methods reveals the tempo of speciation and extinction through time, often in response to major environmental changes (Pyron & Burbrink, 2013; Tietje et al., 2022), but these methods rarely characterize how speciation actually occurs (Leaché et al., 2019; Rosenblum et al., 2012). Consequently, the processes of speciation and maintenance of species boundaries across space, time, and environmental variation are not well known for most taxa (Barton & Hewitt, 1989; Coyne & Orr, 2004; Mayr, 1963; Pyron & Burbrink, 2010). Crucially, the role that environmental variation plays in driving genomic differentiation has not been characterized for diverse assemblages at broad continental scales.

At shallow time‐scales, allopatry is often considered a main driver of speciation and thus the primary cause for extant diversity (Mayr, 1963; Turelli et al., 2001). However, when distinct lineages form across ecotones between biomes, hybrid zones are frequently observed, indicating ongoing interactions representing secondary contact between formerly allopatric populations (Barton & Hewitt, 1989). It is also possible that rapid speciation and adaptation to environments can occur with little evidence of introgression (Roycroft et al., 2024). Alternatively, primary divergence across environmental gradients without isolation (parapatric ecological speciation) may be at play, where adaptive divergence occurs within parts of the genome during the initial process of divergence and—importantly—while species are still connected by gene flow (Feder et al., 2012; Nosil, 2012). Empirical studies increasingly find importance for ecological speciation (Nosil, 2012; Papadopulos et al., 2011), though how widespread it is across continental biomes is unknown. Determining if ecological speciation occurs commonly without geographic isolation (i.e., in parapatry) is paramount to understanding how the genome adapts to distinct environments, reinforcing species boundaries and counteracting collapse via hybridization (Wolf et al., 2001).

Similarly, species boundaries are often recognized without explicit consideration of the environmental and genetic processes that formed those taxa (Burbrink & Ruane, 2021; Jackson et al., 2017). The vast majority of described species are unaccompanied with tests of speciation processes or hypothesized speciation mechanisms in relationship to changing biomes (Padial & De la Riva, 2021; Pyron, 2023). In cases where species are connected via gene flow, delimitation may nonetheless require some understanding of speciation processes like migration and selection (Hey & Pinho, 2012; Petit & Excoffier, 2009; Pyron et al., 2023; Smith & Carstens, 2020).

Correspondingly, some authors have criticized using exclusively genetic methods for species delimitation (Sukumaran et al., 2021; Sukumaran & Knowles, 2017). These criticisms highlight a crucial point: genetically differentiated population structure and even phylogeographic sublineages may exist within species (Pyron, O'Connell, Lemmon, et al., 2022; Singhal et al., 2022) that may not be interpreted as full “species” due to the overall genetic cohesiveness. However, few authors specify how “population structure” or “lineages” differ from species (Burbrink, Crother, et al., 2022; Kizirian & Donnelly, 2008; Pyron et al., 2023). More extreme criticisms suggest that the presence of admixture between lineages demonstrates that reproductive isolation (RI) is incomplete and that subspecies, not species, are being delimited (Hillis, 2020). This criticism fails to acknowledge that the same *kinds* of ontological entities are being delimited (de Queiroz, 2020); species as individuals that are spatio‐temporally bound (Ghiselin, 1974; Hull, 1976). This also highlights the anxiety of delimiting species where gene flow exists (Burbrink, Crother, et al., 2022).

Correspondingly, the presence of gene flow between lineages does not by itself indicate that some degree of RI has not evolved; strong but permeable species boundaries may still be present (Arntzen et al., 2021; Barth et al., 2020; Prager & Wilson, 1975; Price & Bouvier, 2002). Species delimitation using coalescent methods might therefore identify species, but often do not directly address lineage maintenance in most cases (Pyron, O'Connell, Duncan, et al., 2022; Sukumaran & Knowles, 2017). Where genome‐scale data exist in addition to spatial and environmental data, an approach unveiling processes that isolate lineages will help researchers understand how species boundaries are maintained given the existence of lineages that still retain gene flow (Michel et al., 2010; Nosil, 2012; Payseur & Rieseberg, 2016; Wolf & Ellegren, 2017).

Consequently, when geographically distinct phylogeographic lineages have been delimited in preliminary analyses, there are additional expectations required to demonstrate that genomic isolation and thus speciation has occurred. Meeting these requirements can demonstrate that lineages are species and permit integrative hypotheses about the underlying mechanisms of speciation. We outline several of these expectations as follows:

1. Distinct lineages should be discoverable using an integrated approach across multiple data types that link candidate delimitation models to mechanistic hypotheses of speciation (Dayrat, 2005; Pante et al., 2015; Pyron, O'Connell, Duncan, et al., 2022). Here, we use unsupervised machine learning (UML) methods (Derkarabetian et al., 2019) incorporating allelic, spatial, and ecological data (Pyron, 2023). This is the first step to demonstrate that lineage structure is real and discoverable across organismal dimensions.
2. The observed rates of migration or degree of geographic introgression cannot be a passive by‐product of isolation by distance (IBD; Bradburd et al., 2013; Wright, 1943) or processes that do not reflect adaptation by selection (Felsenstein, 1976, 1982).
3. Loci should be significantly correlated with environmental changes regardless of geographic distance, a pattern known as isolation by environment (IBE; Feder et al., 2012; Nadeau et al., 2016; Wu, 2001). This suggests that ecological isolation has played a role in promoting lineage divergence. Where candidate species show IBE, the timing of origin, rates of migration, and mode of divergence should be inferred to provide a context for the process of speciation (Burbrink & Ruane, 2021).
4. If gene flow exists, it should be limited to well‐defined hybrid zones in areas of environmental transition (Burbrink, Gehara, et al., 2021; Harrison & Harrison, 1993; Harrison & Larson, 2014). Exploration of the width and center of the hybrid zone should follow to understand how species boundaries are apparently maintained given gene flow. Genomic clines provide an alternative for understanding the movement of genes through mosaic hybrid zones with admixed individuals where the hybrid zone is not clearly defined spatially (Bailey, 2024; Gompert & Buerkle, 2011).
5. Alleles for a subset of loci should not be freely introgressing but are generally expected to be fixed in either parent, reflecting selection for divergent species boundaries in unique environments (Feder et al., 2012; Souissi et al., 2018). These genes demonstrate the existence of geographical differences that are also historically unique adaptations.

**FIGURE 1:**
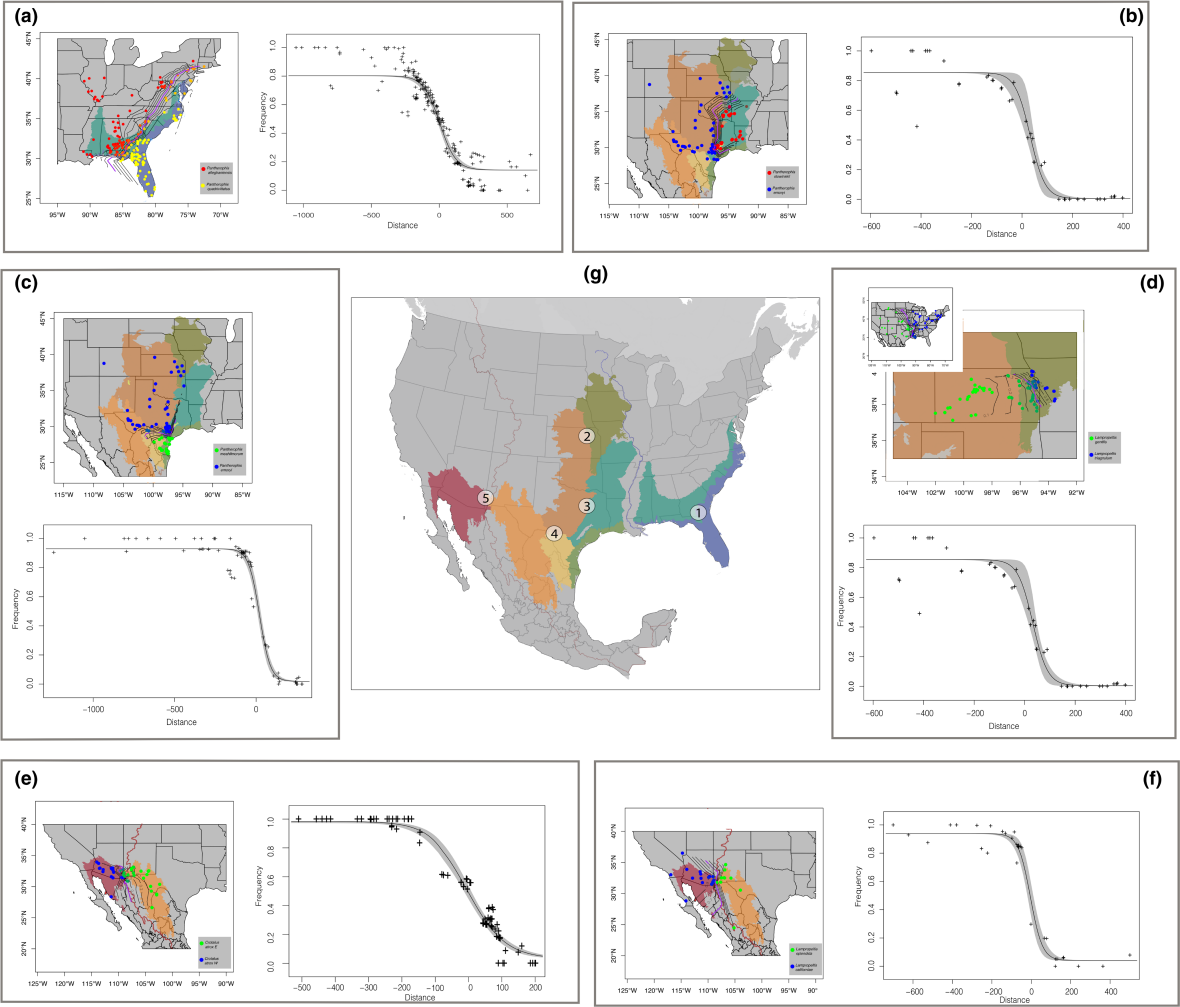
(a) Genomic admixture maps showing the locations of lineages within species pairs given clusters and admixture estimates using the SuperSom (self‐organizing maps) method with interpolated contour lines to reveal the center of the hybrid zone (purple) at the intersections of ecoregions and cline estimates using admixture frequency estimates for *Pantherophis alleghaniensis/P*. *quadrivittatus* (Pa), (b) *Pantherophis slowinskii/P*. *emoryi* (Ps), (c) *P*. *meahllmorum/P*. *emoryi* (Pe), (d) *Lampropeltis triangulum/L*. *gentilis* (Lt), (e), *Crotalus atrox* E/*C. atrox* W (Ca), (f) *Lampropeltis splendida/ L*. *californiae* (Ls). (g) Map showing the intersection between the following ecoregions: 1—southeastern coastal plains (blue) and the southeastern plains (aqua), 2—temperate (olive) and semiarid prairies (tan), 3—southeastern plains/Texas–Louisiana plains (dark green) and the semiarid prairies (tan), 4—semiarid prairies (tan) and Tamailipas–Texas semiarid plain (yellow), and 5—Chihuahuan Desert (orange) and Sonoran Desert (Red) at the Western Continental Divide/Cochise Filter Barrier (brown).

Systems meeting these conditions strongly suggest that environmental differences are maintaining evolutionarily distinct lineages regardless of gene flow in zones of contact. We also note that morphological differences should also arise, however the axis of differentiation (e.g., external morphology, physiology, behavioral) cannot easily be predicted. Here, we incorporate a set of methodological approaches for these five criteria that provide a comprehensive understanding of the formation and maintenance of species that is also ontologically consistent with species as individuals (Hull, 1976). We examine processes of speciation and maintenance of species boundaries across eight lineage‐pairs of snakes in temperate North America that have either been delimited previously as species or are candidates for such designation (Figure 1).

Temperate North America contains numerous connections among distinct biomes ranging from eastern Nearctic and subtropical forests, the Great Plains, and warm and cold deserts (Omernik, 1987). Previous research has demonstrated that many unrelated species show deep lineage structure at the boundaries of these biomes, that include the transition between subtropical/temperate forests at the southeastern coast, temperate forests/grasslands, and Chihuahuan and Sonoran Deserts, suggesting the formation of species in response to associated environmental gradients (Burbrink & Ruane, 2021; Provost et al., 2021; Remington, 1968; Soltis et al., 2006). In particular, wide ranging snake species consistently show deep lineage structure at the intersection of these ecological barriers (Burbrink & Ruane, 2021). However, the mechanisms and processes of ecological speciation are unknown for most of these lineages.

Comparing speciation processes in snakes in temperate North America allows us to define an approach and methodology that yields a set of metrics quantifying the movement of alleles through a hybrid zone relative to environmental change, while assessing the timing and mode of divergence. Studying phylogeography in this way has several consequential benefits. First, it merges the often‐separated research programs of population genetics, species delimitation, and speciation processes in an integrative framework. Second, it clarifies the nature of the entities being delimited and offers a more robust and coherent view of “species” consistent with their ontological individuality as distinct evolutionary lineages. Third, it operationalizes taxonomic revision as an extension of species delimitation while assessing ecological and evolutionary forces responsible for speciation. This synthesis unifies several previously‐disparate lines of thinking regarding ecology, evolution, and systematics, for an integrated understanding of speciation, species delimitation, and taxonomy.

## 2 METHODS

### 2.1 Study systems

Using genome‐scale datasets generated by ourselves and other authors, we examined the following eight species of snakes previously hypothesized to exhibit potentially species‐level divergence with geographic genetic structure satisfying the first criteria that population structure exists (referred to as lineage pairs Table 1; Figure 1): (A) Cornsnakes: *Pantherophis guttatus* (eastern lineages; *Pg*), *Pantherophis emoryi/P*. *meahllmorum* (western lineages, *Pe*), *Pantherophis slowinskii/P*. *emoryi* (western lineages, *Ps*), (B) Pinesnakes: *Pituophis melanoleucus* (eastern lineages; *Pm*), (C) Ratsnakes: *Pantherophis alleghaniensis/P*. *quadrivittatus* (eastern lineages; *Pa*), (D) Milksnakes: *Lampropeltis gentilis/L*. *triangulum* (*Lt*), (E) Kingsnakes: *Lampropeltis splendida/L*. *californiae* (western lineages, *Ls*), and (F) Western Diamondback Rattlesnakes: *Crotalus atrox* (Cochise lineages; *Ca*). Previous research using coalescent methods suggested that most of these lineages are distinct, and could potentially be considered species (Burbrink, Bernstein, et al., 2022; Burbrink, Gehara, et al., 2021; Chambers et al., 2023; Harrington & Burbrink, 2023; Marshall et al., 2021; Myers et al., 2019, 2020; Nikolakis et al., 2022; Schield et al., 2015, 2017). All of the following methods were performed on each lineage pair.

**TABLE 1:** Genomic statistics for eight species pairs of snakes in North America showing the species groups and abbreviations (Abbr), area where divergence occurs, number of individuals (ind) and loci used, genome‐wide fixation indexes (*F*_st_), spatial cline widths (with range), spatial cline centers (with range), number of significant loci assessed using genome clines, average and range for genome cline slope and center (admixture center) for significant loci, *r* ^2^ (adjusted *r* ^2^) for the GDM models (all are significant at *p* = 0.001–0.024), significant non‐spatial GDM variables, number of significant loci assessed using RDA and environmental variables, and number of significant loci using genome scans.

Species group	Abbr	Area of divergence	Ind	Loci	*F*_st_	Spatial cline widths (km)	Spatial cline center (km)	Genome cline loci	Genome cline slope	Genome cline center	GDM *r* ^2^	GDM significant variables	RDA loci	Genome scan loci
*Pantherophis guttatus* (eastern lineages)	Pg	Apalachicola region of Florida	54	10,797	0.05	175 (155–194)	−16 (−23 to −10)	102	21.471 (2.52–59.18)	0.47 (0.40–0.53)	0.42 (0.04)	No variables	486	35
*Pituophis melanoleucus* (eastern lineages)	Pm	Apalachicola region of Florida	38	2689	0.04	283 (235–328)	−9 (−24 to 5)	23	25.91 (5.24–78.35)	0.68 (0.61–0.73)	0.64 (0.11)	No variables	66	101
*Pantherophis alleghaniensis/P*. *quadrivittatus*	Pa	Southeast Coast Plains and Southeastern Plains	206	846	0.08	236 (163–340)	4 (−0.2 to 8.1)	26	7.80 (1.49–47)	0.52 (0.45–0.63)	0.17 (0.08)	Bio15, Bio19, Bio5, Bio8	34	8
*Pantherophis slowinskii/P*. *emoryi*	Ps	Southeastern plains/Texas–Louisiana plains and Semiarid Plains	64	6993	0.22	3 (33–38)	−18 (−19 to −16)	58	4.11 (1.61–46.29)	0.52 (0.48–0.55)	0.29 (0.10)	Elevation, Bio1, Bio3, Bio9	724	198
*Pantherophis emoryi/Pantherophis meahllmorum*	Pe	Semiarid plains and Tamailipas‐Texas semiarid plains	76	6993	0.22	128 (120–180)	20 (17 to 23)	75	3.02 (1.64–33.08)	0.54 (0.44–0.64)	0.28 (0.10)	Bio7, Bio8	755	113
*Lampropeltis gentilis/L*. *triangulum*	Lt	Temperate and Semiarid Plains	85	3246	0.1	211 (202–219)	−11 (−25 to −8)	41	15.01 (2.32–78.49)	0.42 (0.41–0.44)	0.22 (0.07)	Elevation, Bio3	236	87
*Lampropeltis splendida/L*. *californiae*	Ls	Cochise Filter Barrier	38	7878	0.27	109 (18–217)	−2 (−7 to 4)	58	12.786 (1.97–40.07)	0.42 (0.26–0.54)	0.54 (0.14)	Bio18	387	440
*Crotalus atrox* (*Cochise lineages*)	Ca	Cochise Filter Barrier	44	7951	0.18	88 (17–25)	34 (28 to 40)	37	15.62 (2.20–54.55)	0.72 (0.60–0.88)	0.40 (0.07)	Bio19	904	198

### 2.2 Lineages and structure

Our first criterion for understanding speciation was identifying groups across an integrative range of data types. To determine lineage structure for all downstream analyses, we used three approaches: an UML, self‐organizing maps (SOMs) method (see below), discriminant analysis of principal components (DAPC; Jombart et al., 2010), and TESS3r (Caye et al., 2016). For SOMs, we incorporated geographic and environmental data used to delimit populations and assess admixture. Bioclim environmental variables describing temperature and precipitation (bioclim 1‐19), elevation, net primary productivity (mean monthly MODIS normalized difference vegetation index; NDVI), and the percentage of woody plants (datasets downloaded from http://www.paleoclim.org/ and https://github.com/rebeccalpowell/grassmapr) were extracted for each genetic sample location using the R package raster (Hijmans et al., 2014). We assessed correlation among each of the bioclim variables and removed variables correlated with *ρ* > 0.90 for each species pair.

DAPC is a model‐free method using *K*‐means to sequentially estimate the minimum number of genetic clusters and assigns individuals into those clusters without prior group identification. Using the adegenet package (Jombart, 2008) in R (R Core Team, 2010), data were transformed using principal component analysis (PCA) with 150 axes and maximum of 20 groups. All discriminant axes with eigenvalues >1% of the sum were retained. To reduce the probability of PCA generating arbitrary groupings, we took the difference between actual and randomized cluster assignments and calculated the optimal number of axes out of 150 to eliminate bias. With these optimal number of axes, we reran DAPC and chose the number of groups with the lowest BIC (Bayesian information criterion) value. We then cross‐validated the group results using 90% training and 10% test datasets and estimated the average predicted success for each group. We also noted genes showing contributions to the principal components of DAPC >99% of all loci for comparison with other methods (see below).

We also generated individual ancestry coefficients in a geographic context using the graph‐based nonnegative matrix factorization algorithm (Frichot et al., 2014) in the TESS3r package in R (Caye et al., 2016). With geographic coordinates for all samples, we estimated *K* = 1–10 with 200 iterations. We calculated differences in root mean squared error (RMSE) to predict where increasing values of *K* yielded diminishing values of RMSE. This method always produces lower values with increasing *K*‐values, however, after a certain threshold *K*‐values fail to generate geographically meaningful groups. For each grouping, we also estimated the fixation index (*F*_st_) for each lineage pair. *F*_st_ was estimated using the function pairwise.WCfst in the R package hierfstat (Goudet, 2005).

Finally, we performed integrative species delimitation using SOMs. Most approaches for genetic‐based species delimitation rely solely on molecular data (Leaché et al., 2019; Yang & Rannala, 2010), or are limited to a few traits under a restrictive parametric model (Solís‐Lemus et al., 2015). Even recent UML methods have typically been limited only to allelic data (Derkarabetian et al., 2019, 2022).

Here, we use the expanded SuperSOMs method incorporating UML method for species delimitation. This uses the self‐organizing or “Kohonen” maps (SOM) introduced by Pyron (Pyron, 2023) implemented in the “delim‐som” package and relying on the “kohonen” package (Wehrens & Buydens, 2007) in R for model optimization. For each taxon, we had allelic, spatial, and climatic data as described above. We estimated 3‐layer SuperSOMs incorporating each of these datasets independently, without a 4th trait layer. We used 100 replicates learned over 100 steps with a learning rate of [0.5, 0.1]. We converted allelic data to frequencies per locus and min–max normalized the spatial and climatic data. For each taxon, we estimated a SuperSOM grid with hexagonal cells and a Gaussian neighborhood function, with dimensions of 5 times the square root of the number of individuals (i.e., rows in the input layers), a common rule‐of‐thumb (individual clustering estimates for each taxon are given in, Figures [Link], [Link], [Link], [Link], [Link], [Link], [Link]). We also estimated the species coefficients (i.e., the “admixture” or “ancestry” values estimates for each specimen across all layers) to compare with the individual ancestry coefficients estimated using “TESS3r” (Caye et al., 2018). This allowed us to compare the decisiveness of the combined datasets versus the genetic data alone in assigning individual specimens to genetic lineages or species.

### 2.3 Demographic modeling

The demographic history of phylogeographic lineages contains multiple parameters that are relevant both for inferring their evolutionary history and the mode of speciation as well as making ontological judgments as to their status as species versus population structure (Burbrink, Crother, et al., 2022; Jackson et al., 2017; Leaché et al., 2019). The parameters include divergence times, population‐size changes, and migration rates, for which we optimized demographic models based on the site‐frequency spectrum (SFS) using the genetic algorithm “GADMA” (Noskova et al., 2020, 2022) based on the “moments” engine (Jouganous et al., 2017). For most comparisons, this involved estimating a 2‐population model, although for the (Marshall et al., 2021) dataset for the western lineages of Cornsnakes (*Pantherophis emoryi/P*. *meahllmorum/Pantherophis slowinskii*) we fit a 3‐population model to the western lineages alone. We first down projected the VCF files to 2‐ or 3‐dimension SFS using “easySFS” (https://github.com/isaacovercast/easySFS; Gutenkunst et al., 2009). Down‐projecting based on the informal criterion of allelic sample size that maximized the number of segregating sites per lineage typically resulted in SFS that were too large and sparsely populated for effective inference in preliminary trials. Consequently, we took a more conservative approach and chose the dimensions at which the second‐order increase in the number of segregating sites was less than or equal to 0. For example, if a 2‐dimensional SFS of [5, 5] yielded 100 segregating sites, [6, 6] yielded 200, and [7, 7] yielded 250, we chose [6, 6] rather than [7, 7].

Germline mutation rates (per site, per generation) are poorly known in snakes, with only one species (*Thamnophis sirtalis*) estimated at 2–3 × 10^−8^ (Bergeron et al., 2023). Furthermore, direct genomic measurements are potentially an order of magnitude higher than long‐term substitution or fixation rates. We therefore followed recent authors in using a value of 6 × 10^−9^ for reptiles (Burbrink, Bernstein, et al., 2022; Harrington et al., 2018; Myers et al., 2020). Similar rates of ~1–4 × 10^−9^ have been used by several authors for analyses of butterflies, crustaceans, mollusks, and rodents in GADMA (Agwamba & Nachman, 2023; Amador et al., 2022; Pazhenkova & Lukhtanov, 2023; Peluso et al., 2023). To account for plausible empirical variation in substitution dynamics, we also ran analyses with rates of 2 × 10^−9^ and 1 × 10^−8^ to provide a range of parameter estimates.

For smaller‐bodied taxa, we used a consensus generation time of 2.5, and 3.5 years for larger species (Ernst & Ernst, 2003). We took the full sequence length retaining all SNPs (not just unlinked) to generate the SFS from the original assemblies obtained from the initial publication or directly from the authors. Specifying mutation rate and sequence length and including generation time allows re‐scaling to thousands of years. We allowed asymmetric migration, specified unlinked SNPs with no outgroup, and enforced an initial and final structure with one time‐period per divergence (i.e., 1, 1 or 1, 1, 1). We used 100 optimizations of the genetic algorithm in GADMA for each of the three rates to arrive at the final models given initial settings, runtime conditions, and parameter estimates (Table S1).

### 2.4 Genome scans

To understand which loci showed evidence of selection given that apparently real lineage structure exists—satisfying criterion 5—we used the R package pcadapt (Luu et al., 2017). This method first estimates population structure using PCA and generates admixture estimates for each individual. We checked these estimates with those discussed above and found the similar population structure for PC1 and PC2. Then, given the number of principal components required to predict population structure (here always *K* = 2 for all pairwise comparisons), we used the pcadapt function which regresses each locus with these principal components to generate a *Z* score. A test statistic based on the Mahalanobis distance of each SNP to the mean was produced. These squared distances when divided by a genomic inflation factor were chi‐square distributed with *K* degrees of freedom and used to estimate a *p*‐value. We used the Benjamini–Hochberg procedure to correct *p*‐values given a false discovery rate (Benjamini & Hochberg, 1995) and sorted these for significantly selected loci below 0.1.

### 2.5 Environmental‐genomic relationship

To determine if the environment affects genetic distance while accounting for geographic distance we used the generalized dissimilarity modeling (GDM; Ferrier et al., 2007; Fitzpatrick & Keller, 2015) approach in the R package gdm (Fitzpatrick et al., 2022) following Mokany et al., (2022). We used uncorrelated environmental data (same as described above for SOM). This allowed us to understand if genetic distance was significantly associated with environmental changes suggesting a strong role of IBE satisfying criteria 2 and 3. We calculated linear geographic distances and uncorrected genetic distances in base R to simultaneously test the effect of geographic and environmental distances on genetic distances using the uncorrelated environmental and elevation variables described above. We calculated pairwise distances among samples for each variable and determined if environmental distances plus geographic distance significantly predicted genetic distance. We also estimated importance of each variable in the GDM model.

### 2.6 Spatial clines

We determined if a well‐defined hybrid zone exists at environmental transitions satisfying criterion 4. Using admixture proportions estimated from the TESS3r analysis interpolated over geographic area, we first predicted the general region and geographical center of the hybrid zone using the R package akima (Akima et al., 2016). This objective measure provided the area of 50% admixture (i.e., the center of the cline or hybrid zone) used for examining spatial clines. We calculated the geographic gradient of genomic differences between spatially adjacent lineages by estimating the steepness of these differences to generate the width of the cline.

Here, steeper clines have relatively narrower widths and may be the result of selection on hybrids or parental species in an environmental cline. These two‐dimensional samples mapped over space were reduced to a single dimension appropriate for spatial clinal analyses in the R package HZAR (Derryberry et al., 2014) by taking the geographic distance between each sample and the center of the hybrid zone estimated from akima, and assigned a positive or negative sign to each distance given the orientation of each individual to the center line of the admixture cline. To estimate width and center we used the admixture proportions for each sample and distances to the spatial cline center to fit the following five sigmoidal clinal models in HZAR using AICc under the Gaussian cline model: (1) no tails, (2) right tail only, (3) left tail only, (4) mirrored tails, and (5) both tails estimated independently (see Derryberry et al., 2014). In the package HZAR, we ran MCMC chains for 5 × 10^6^ generations and thinned by 5 × 10^3^ generations. We assumed stationarity when the estimated sample sizes (ESS) >200 in the R package CODA for width and cline center (Plummer et al., 2006). For each locus, we calculated spatial cline width and center using HZAR given the individual allele frequencies at that locus. We used the same individual distance and orientation to the admixture center line and ran models as described above. For each locus we also estimated fixation where each allele occurs in >80% of each cline tail (5% of the samples) representing parental lineages. We estimated both spatial cline width and center for each locus.

### 2.7 Genome clines

Because the location and nature of spatial clines may be biased when the size and type of hybrid zone changes over large distances, exists as a mosaic, or is the result of complex historical interactions, we also examined genome clines (Gompert & Buerkle, 2011; Szymura & Barton, 1986). This method has the advantage of estimating selection (or drift) against allelic introgression through admixed individuals regardless of space. This eliminates reliance on a dispersal/selection balance and permits researchers to identify parameters that affect introgression without spatial assumptions like having a smooth, sigmoidal spatial cline. We used the R package GG Hybrid (Bailey, 2024) to calculate steepness of the cline on a graph where the abscissa represents the hybrid index between parental lineages and the ordinate represents locus‐specific allele frequencies. Significantly steep genome clines (*ν* > 1) indicate selection (or drift) on locus introgression. Additionally, the center of the genome cline (*υ*) was also calculated and indicates how far alleles introgress between lineages, assuming that the center of the cline represents 50% admixture and *u* = 0.5, where there is no bias in introgression into one parent or the other. When *v* = 1, this indicates no deviation from the genome‐wide average in frequency of allele copies originating from a single parental lineage.

To estimate the genome‐wide hybrid index we chose parental lineages by selecting individuals that show >80% of the genome originating from one parent or the other from the TESS3r ancestry coefficients. We estimated the genome‐wide hybrid index with 5000 iterations and a burn‐in of 1000 based on recommendations from the author (Bailey pers.comm.) using the function esth. Results from 2 to 4 runs were compared to ensure the same outcome for each lineage pair. We estimated cline steepness and center for each locus using the function ggcline with 5000 iterations and a burn‐in of 2000 (Bailey pers.comm.). From this we estimated significantly steep loci (*ν* > 1) and the center (*υ*) for each locus given the genome‐wide average.

### 2.8 Locus‐environment interactions

To understand if specific loci are under selection given changes in environment, we used a redundancy analysis (RDA; Forester et al., 2018). This determines how loci covary relative to a multivariate assessment of environmental data (Rellstab et al., 2015). The environmental data are described above and represent uncorrelated bioclim, woody plant, NDVI for each month, and elevational data. Here, missing allelic data were imputed from the average value for each locus. We ran the rda function in vegan (Dixon, 2003) that performs multilinear regression on genetic and environmental data yielding a matrix of fitted values. These values were used to produce canonical axes of linear combinations of the environmental variables. We generated an *r* ^2^ value for the model which shows how much variance environmental data predicts. We assessed the significance of the model using the anova.cca function in vegan.

To determine which SNPs represent potential adaptation to environmental variables we extracted SNP loadings from our RDA model and took those loci occupying 2.5 standard deviations in the tail (*p* < 0.015). We removed SNPs that were duplicated across more than one RDA axis and assessed which environmental variable was most strongly correlated with these significant loci. Finally, we used Venn diagrams to examine the overlap of significant loci associated with (1) delineating lineages using DAPC, (2) spatial clines, (3) genome clines, (4) genome scans, and (5) environmental interactions from RDA. With the subsets of loci overlapping among these metrics, we then assessed lineage structure over space and *F*_st_. We proposed that those reduced‐number of loci that retain spatial‐lineage structure similar to the entire SNP dataset but with higher *F*_st_ values strongly indicate that selection against introgression is maintaining species boundaries.

## 3 RESULTS

### 3.1 Lineage structure

All methods including SuperSOM, DAPC, and TESS3r showed each of the eight a priori species pairs are generally best fit as two distinct spatial lineages meeting criterion 1. The exceptions were *Pg* and *Pm*, where each was best represented by *K* = 1, but at *K* = 2 still showing geographic groupings (Figure 1; Figures [Link], [Link], [Link], [Link], [Link], [Link], [Link]). An additional exception occured for *Pm* in the SuperSOM analysis, which estimated 3–5 genetic clusters of genetic variation admixed across nearly the entire range of the broader species *Pituophis melanoleucus*. We interpreted this as *K* = 1 in the broad sense of a single lineage reflecting local geographic genetic structure without parental lineages fixed in any biogeographic region. Admixture estimates between SOM and TESS3r were correlated (*ρ* > 0.91; Figure S8).

The species‐delimitation analyses using three‐layer climate‐based SuperSOMs were consistent with the clustering or similar results from previous papers (Figures [Link], [Link], [Link], [Link], [Link], [Link], [Link]). Our results supported two species of Milksnake (*Lampropeltis triangulum* and *L*. *gentilis*), four species of Cornsnake (*Pantherophis guttatus*, *P*. *emoryi*, *P*. *meahllmorum*, and *P*. *slowinskii*), two species of eastern Ratsnake (*P*. *alleghaniensis* and *P*. *quadrivittatus*), two species of western Kingsnake (*L*. *californiae* and *L*. *splendida*), and two species of Western Diamondback Rattlesnake (*Crotalus atrox*) which are currently known only by their Eastern and Western lineage designations. These clustering results were supported at ~100% for all taxa, suggesting very little variation in the inferred numbers of *K*. Similarly, allelic frequencies dominated the model output for all taxa, and the impact of climate was greater than that of space in all instances. Notably, SuperSOMs have previously been shown to be relatively conservative in delimiting species (Pyron, 2023), suggesting that these lineages had a high likelihood of species‐level distinctiveness in comparison to other well‐established taxa.

Accordingly, the SuperSOM analyses did not delimit the insufficiently divergent Central and Eastern Cornsnake lineages of (Myers et al., 2020), a result corroborated by the GADMA2 demographic estimates of very high migration rates (see below). Similarly, this analysis supported a single species for all Cornsnakes samples from west of the Mississippi River sequenced by Myers et al. (2020), which would be *P*. *emoryi* as concluded by (Marshall et al., 2021). However, unlike the densely sampled eastern region, Myers et al. (2020) included relatively few and sparsely distributed samples from the western populations assigned to *P*. *slowinskii* and *P*. *meahllmorum*, and SuperSOMs were suspected to have a difficult time delimiting poorly sampled lineages (Pyron, 2023). Corroborating this, the SuperSOM analyses of the Marshall et al. (2021) dataset strongly supported all four species, as noted above. The contrastingly incomplete sampling regimes of Myers et al. (2020) and Marshall et al. (2021) and their attendant inconsistencies in species delimitation highlight the need for geographically dense sampling not just near contact zones (Chambers et al., 2023) but also range wide.

### 3.2 Demographic modeling

**FIGURE 2:**
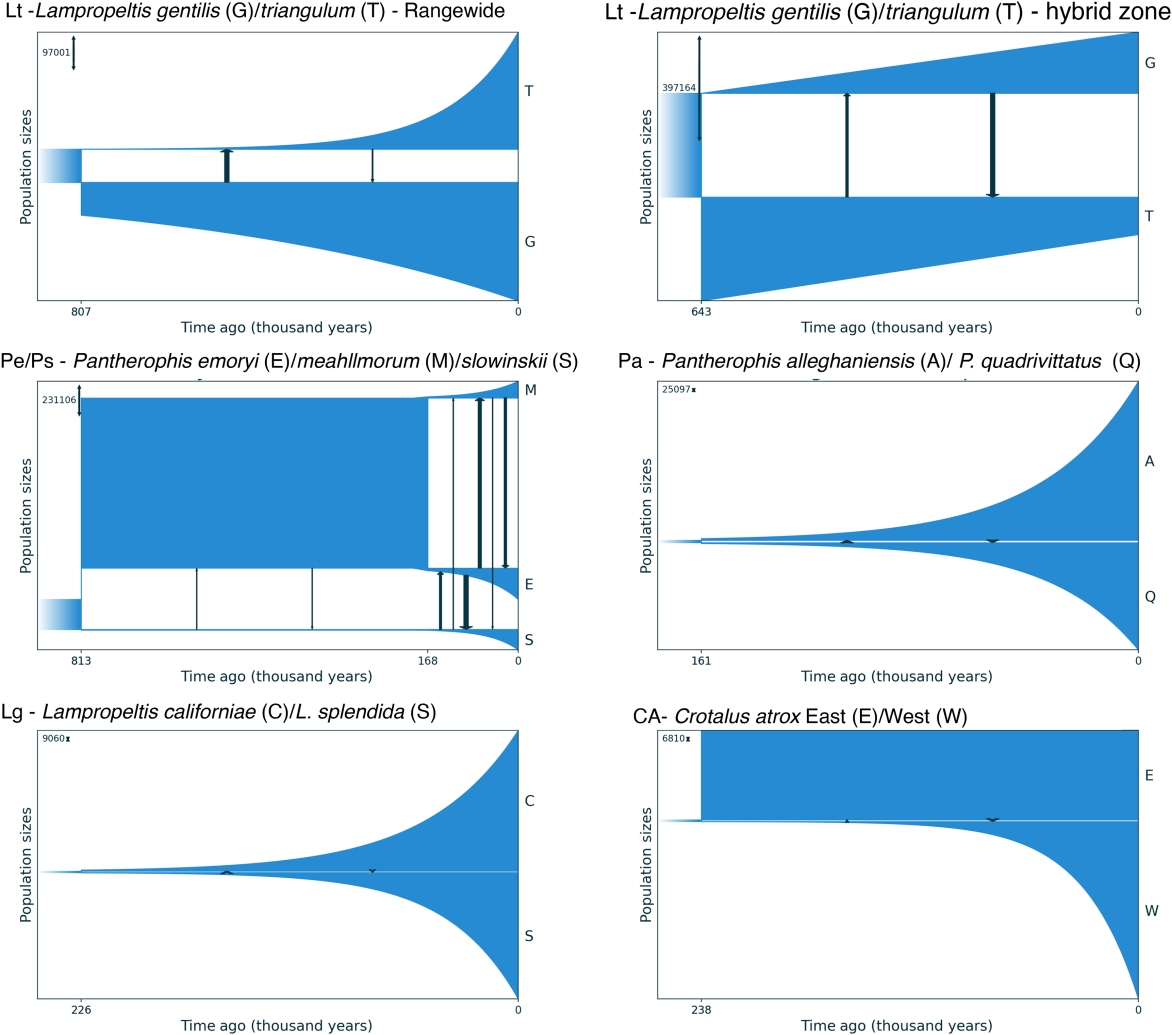
Best‐fit isolation and migration models using GADMA2 for all species pairs showing distinct clusters from the SuperSOM analysis. Time of divergence and changes in population sizes (width of blue lines) and migration rates (black arrows) are shown for all species pairs.

To place the origins of these groups in a historical context, we estimated divergence time, effective population size, and migration rate for each set of lineages revealed concordant patterns supporting or rejecting their species‐level distinctiveness (Figure 2, Table 1, Table S2). For the Milksnakes, the divergence between *L*. *gentilis* and *L*. *triangulum* dated to ~600–800 Ka using the datasets of (Chambers et al., 2023) and (Burbrink, Bernstein, et al., 2022). Migration rates estimated using the former dataset were very high (~9–20 migrants per generation), but their sampling primarily comprises admixed specimens from the hybrid zone. Chambers et al. (2023) did not explicitly estimate ages or migration rates. The full sampling from Burbrink, Bernstein, et al. (2022) predictably decreased migration estimates by an order of magnitude when including pure parental populations from across the range of both species. This yields rates <1 from *L*. *triangulum* into *L*. *gentilis* (~0.37 migrants per generation) as expected when genetic divergence outpaced gene flow during speciation. Migration in the reverse direction from West to East (*L*. *gentili*s in *L*. *triangulum*) was higher (~1.9 migrants per generation). Burbrink, Bernstein, et al. (2022) estimated slightly older ages (~1.4–3.6 Ma) and similar migration rates (~0.45–0.82) using PipeMaster (Gehara et al., 2020) which did not take into account population‐size changes, a factor known to heavily impact demographic‐parameter estimates (Momigliano et al., 2021) and potentially alleviated here in our GADMA2 models. As a corollary to the sampling issues noted above for Cornsnakes, Chambers et al. (2023) focused on sampling in contact zones, potentially obscuring the more accurate estimation of migration rates across the range when including pure parental populations of each Milksnake species.

The western Cornsnake lineages were estimated to be a similar age to the Milksnakes at ~813 Ka (755–3550 Ka) for the origin of *P*. *slowinskii* and ~168 Ka (131–580 Ka) for the divergence between *P*. *emoryi* and *P*. *meahllmorum*. Migration between some of these species was also similarly low (<1 migrant per generation between *P*. *slowinskii* and *P*. *meahllmorum*), though modestly higher (~4–9) between most other pairwise combinations of species and relatively high (~7–13) from *P*. *emoryi* into *P*. *slowinskii*. The distinctiveness of these species in the face of such apparent but spatially proximate introgression (see below) suggests that other ecological or genetic mechanisms must be at play to maintain their evident and distinct evolutionary trajectories over time. The Central and Eastern Cornsnake lineages of Myers et al. (2020) date to ~1800 Ka (880–5100 Ka), but with very high migration rates of ~7 migrants per generation from East to Central and ~20 in the opposite direction. Combined with the species‐delimitation results from the SuperSOM analysis, this suggested that this relatively ancient geographic genetic diversity represented only local population structure, rather than full species. Myers et al. (2020) estimated the age of this divergence to be younger at ~448 Ka (92–912 Ka) and similarly high migration rates of up to ~26 migrants per generation between them. Those estimates used fastsimcoal2 (fsc2) and therefore may also be affected by static effective population sizes in the models. Given the limited sampling of *P*. *emoryi*, *P*. *meahllmorum*, and *P*. *slowinskii* in the dataset of Myers et al. (2020), we did not model their demography separately using these data.

The remaining species (eastern Ratsnakes, western Kingsnakes, and Western Diamondbacks) each formed a cluster of younger, late Pleistocene divergences ~161–238 Ka and subsequent exponential increases in effective population size, suggested the impact of recent glacial cycles and climatic refugia on their origin. The divergence between *P*. *alleghaniensis* and *P*. *quadrivittatus* dates to ~161 Ka (97–483 Ka), with modest levels of migration in each direction of ~1.4 migrants per generation. These estimates were lower (both age and migration) than the previous PipeMaster analyses of these data (Burbrink, Gehara, et al., 2021). Similarly, *L*. *californiae* and *L*. *splendida* dated to ~227 Ka (136–680 Ka) with near‐zero migration in either direction of ~0.03–0.08 migrants per generation. Previous authors did not estimate these parameters using this dataset (Myers et al., 2019) but Harrington and Burbrink (2023) inferred a much older time of divergence for these lineages (~1.94 my) using fastsimcoal2 (Excoffier et al., 2021). Across the same region (the Cochise Filter Barrier between the Chihuahuan and Sonoran Deserts in southwestern North America), the Western and Eastern lineages of *C*. *atrox* dated to ~238 Ka (143–718 Ka) with low migration in either direction of ~0.03–0.1 migrants per generation.

### 3.3 Environmental structure

**FIGURE 3:**
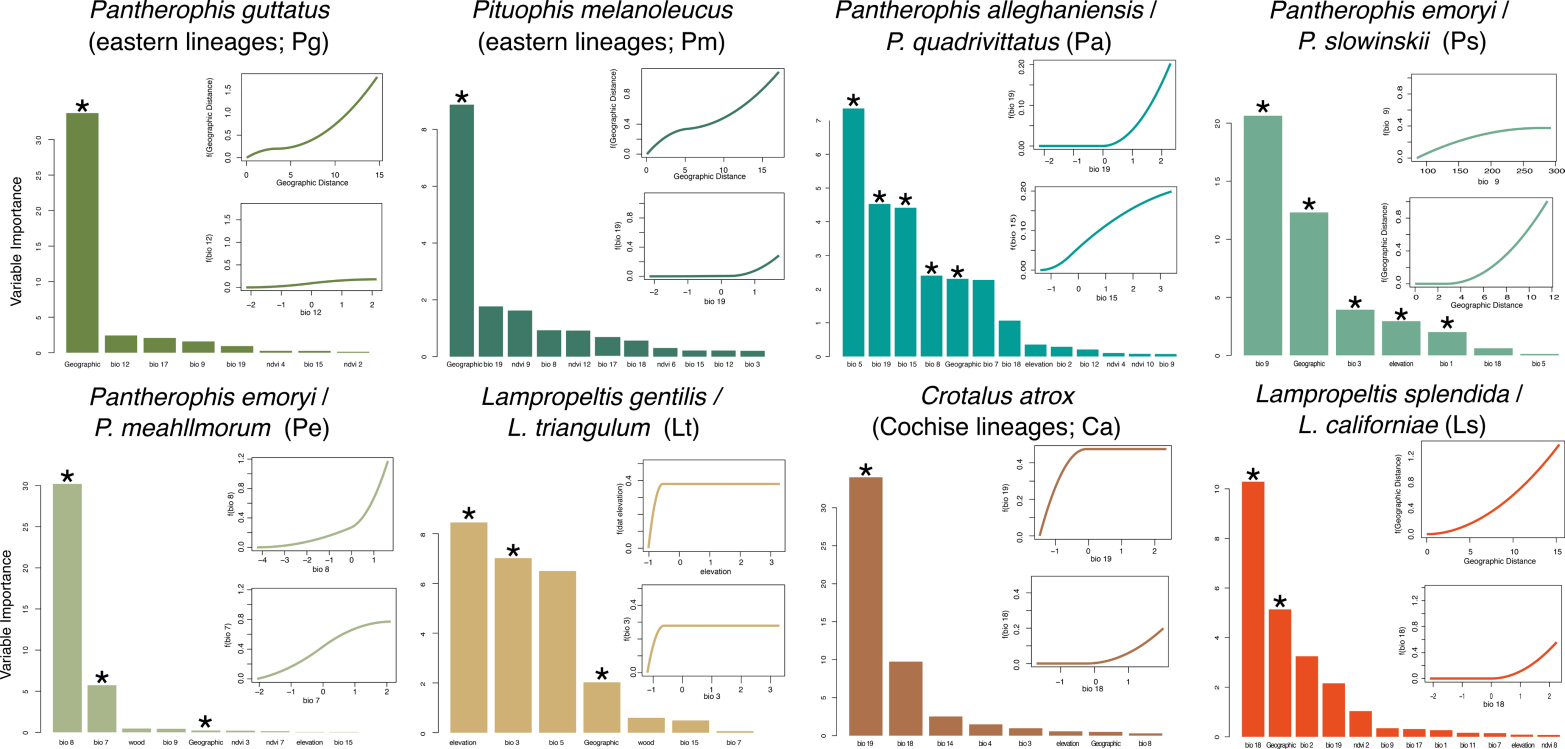
Histograms showing variable importance from general dissimilarity models (GDMs) for each species pair. Asterisks above variables indicate significance.

Significant environmental structuring was predicted using GDM while accounting for geographic space in six taxon pairs (*p* < 0.07) excluding *Pg* lineages and *Pm* lineages (Figure 3, Table 1). The other six taxon pairs therefore meet criteria 2 and 3, showing that numerous distinct genes have responded strongly to environmental changes despite geographic distance. Given significance and variable importance, Bioclim variables associated with precipitation and/or temperature had a large effect on structuring the remaining lineages.

### 3.4 Clines

For all taxa, we determined the center of the cline interpolating admixture over space (Figure 1). Using HZAR (Derryberry et al., 2014) we estimated cline widths and centers (Table 1), with the lowest average width of 35 km for *Ps* and highest width of 283 km for *Pm* (Table 1). Cline centers were between −16.83 and 33.33 km from the predicted center of the cline. Examining loci with cline widths lower than estimates for admixture widths, we found as few as 0 loci in *Pg*, *Pm*, and *Pa*, but as many as 91 loci in *Ls* (Table 1; Figure S9). This indicated that for most of these lineage pairs a well‐defined hybrid zone exists at environmental transitions (criterion 4). Also combining data for *Lt* (Burbrink, Bernstein, et al., 2022; Chambers et al., 2023), we showed the importance of sampling both the hybrid zone and much of the parental ranges to better infer the steepness of the cline (Figure S10).

**FIGURE 4:**
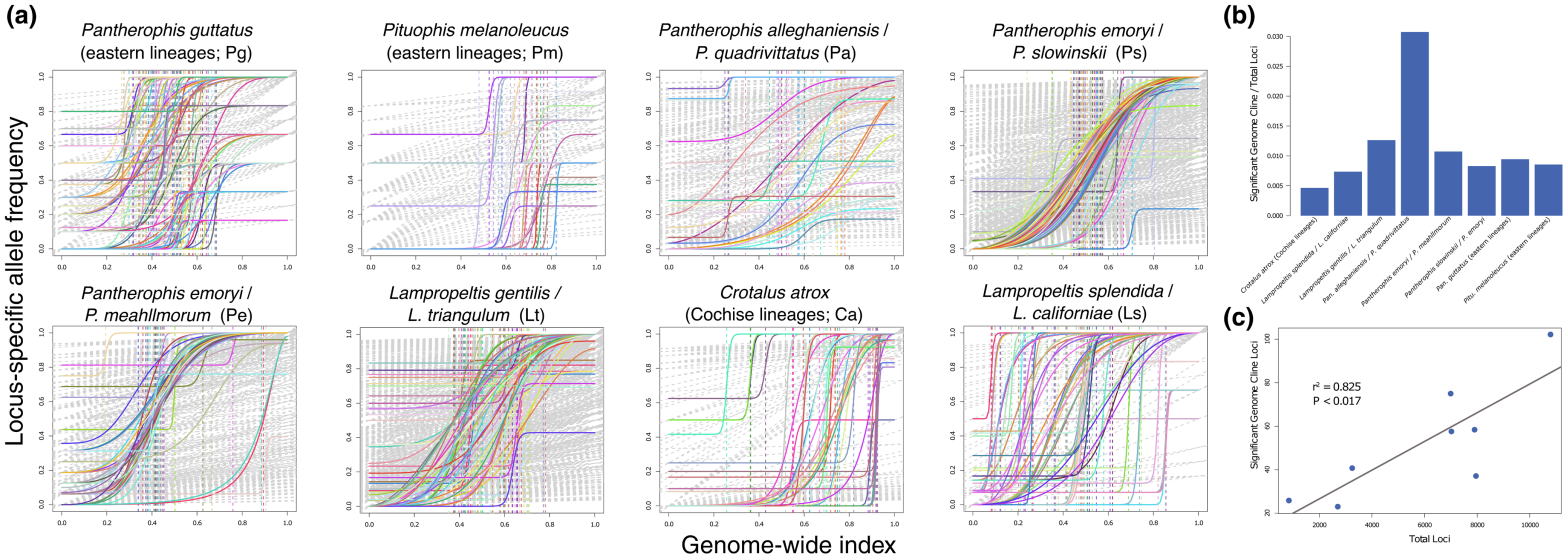
(a) Genome clines for all loci for each species pair. Gray lines indicate loci not showing a significant slope (v) > 1. Colored solid lines indicate loci with significant slope and colored dashed lines indicate the location along the genome‐wide hybrid index. (b) Proportion of significant genome cline loci as a percentage of all loci sampled for each species pair. (c) Correlation between the total number of loci sampled across all pairs and the number of significant loci discovered using the genome cline method.

Because spatial clines may be inaccurate for assessing selection or drift of alleles between lineage pairs in a geographic mosaic, we also estimated genome clines. These cline estimates with slopes significantly larger than *v* = 1.0 (average per lineage pair ranged from *v* = 3.0 to 25.9) varied among taxa but were present in a minimum of 23 loci in *Pm* and a maximum of 102 loci in *Pg* (Figure 4), indicating support for criterion 5. The number of significant genome cline loci were correlated with the number of loci sequenced (*ρ* = −0.82; *p* = 0.01). Cline centers over all significant loci for each lineage pair averaged from 0.42 to 0.68, indicating most loci do not show directional movement of alleles far into one lineage or the other past the admixed center of 0.50, though variance and ranges are high for some taxa.

### 3.5 Locus‐environmental interactions

Seven of the eight lineage pairs had more than 100 loci correlated with environmental variables across all four categories (bioclim, NDVI, woody, and elevation), strongly suggesting that adaptations were detectable over these widely changing environments (criterion 3). *Pa* had only 33 significant loci (though this taxon pair had only 846 loci total; Figure S11). Certain taxa showed most loci dominated by one or a few variables including *Pg* (Bio 3; 50%) and *Ls* (Bio 14; 98%). Using genome scans to assess which loci show evidence of selection, we found that between 8 and 440 loci were strongly selected between lineages (Table 1). This fits criterion 5 suggesting that for most lineage pairs, alleles from subsets of loci were not freely introgressing.

### 3.6 Overlap among selection metrics

**FIGURE 5:**
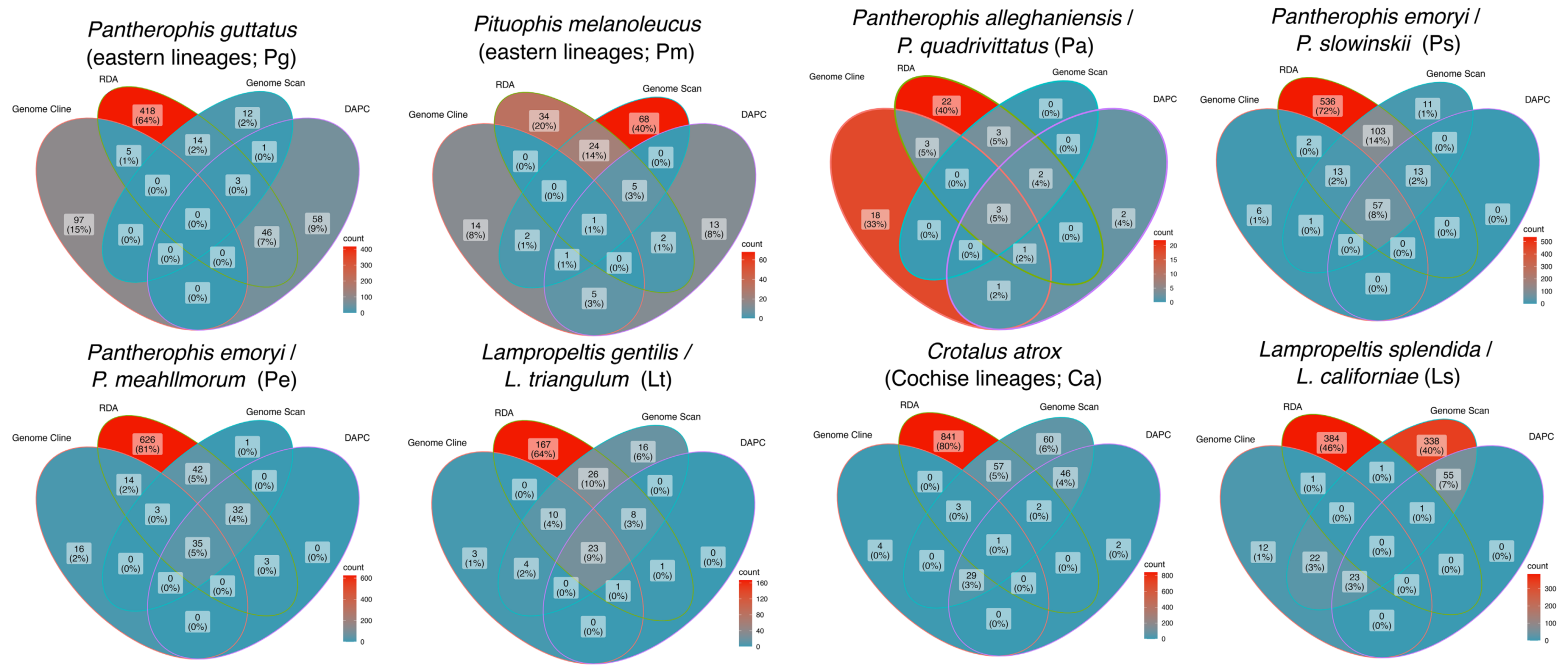
Venn diagrams showing the number (and percentage) of loci that overlap among the following categories: RDA—environmental‐locus correlations, significant genome clines, significant genome scans, and 1% of loci showing contributions to the principal components for identifying clusters in DAPC >99%.

Overlap among spatially restricted, genomic outlier, DAPC, and RDA loci ranged widely given species pairs, from the greatest overlap among metrics in *Ps* at 57 loci to the collapsed *Pg* at zero loci (Figure 5). Data from spatial clines were excluded because they often failed to detect arbitrarily low widths. Genome scans, genome clines, and DAPC showed more overlap with each other than RDA loci. Among those three former metrics (excluding RDA), we found up to 57 loci (0.81% of sampled SNPs) overlapping in *Ps* and as low as only 2 loci (0.074%) in *Pm*. With these overlapping loci responding to some form of selection, we found that all of these captured the same geographic structure as using the entire genomic dataset when rerunning DAPC (*K* = 2; Figure S12). This indicated that those loci responding to selection at the hybrid zone between species were also those that identify the lineages geographically and help support criteria 4 and 5.

## 4 DISCUSSION

We find robust support for the buildup of terrestrial biodiversity at continental scales through ecological speciation occurring across the boundaries or transitions between major biomes. We present evidence from spatial and environmental analysis of genomic divergence that suggests strong differentiation of emerging lineages due to environmental selection at ecotones and species boundaries maintained despite gene flow at hybrid zones of varying width. This results in a diversity of species overlapping at the intersection of environmental boundaries across a continuous spatial landscape, where genomic differentiation maintains lineage identity despite hybridization. Within major biomes, such as eastern temperate forests, geographic genetic structure of populations yields incompletely diverged lineages in *Pg* and *Pm* that are correspondingly not recognized as species.

In snakes, it appears that either ecological speciation or secondary contact is a main driver of the buildup of biodiversity in young taxa across distinctly different biomes in North America. Pure allopatric speciation with no gene flow when in contact was not apparent for any of the taxa examined here. Furthermore, we demonstrate that when lineages are detected, species can be delimited confidently when boundaries among species limits are maintained by selection on particular loci. This research underscores that strict allopatry may not be the driving force behind speciation across biomes and suggests that alternative mechanisms require examination across communities of organisms to understand the dominating processes of speciation across continents.

We demonstrate that when lineages can be identified genetically, geographically, and environmentally using SNP‐based hierarchical clustering techniques, they also tend to show selection at key loci identifying those lineages, even with zones of high admixture (Figures 1, 4, and 5). All but two lineage pairs show environmental variables related to lineage structure over space at key biogeographic transitions. Both *Pg* and *Pm* are best predicted to only show a single lineage across the Apalachicola/Appalachian region, without significant environmental barriers structuring these lineages. Consequently, the observed genetic structure in those taxa does not appear to reflect diagnosable IBE (Wang & Bradburd, 2014). For the remaining lineage pairs, the same loci predicted to show turnover in genotypes through admixed individuals are those showing signatures of selection using genome scans occurring at well‐defined hybrid zones between contrasting environments, thus fitting speciation criteria 1–5. These results strongly indicate that lineages with hybrid zones and deep divergences are maintained by selection against alleles when connected geographically, rather than representing weakly divergent local adaptations or population structure within a single species.

In contrast, strongly supported speciation events at biogeographic barriers across North America all show evidence of IBE. Our historical demographic estimates indicate these lineages all originated in the mid‐to‐late Pleistocene and have remained distinct for 34,000–325,000 generations despite moderate gene flow (Figure 2). Genetic variation among these lineages is correlated with different combinations of environmental variables over geography (Figure 3). However, the *Pg* and *Pm* lineage pairs at *K* = 2 were likely a spurious result caused by IBD. This is similar to other taxa occupying the Pliocene embayment of the Apalachicola region in the US, with few interpreted as species‐level divergences (Burbrink et al., 2000; Church et al., 2003; Randazzo & Jones, 1997; Soltis et al., 2006; Soto‐Centeno et al., 2013).

All other species pairs demonstrate a pattern of IBE. For example, we see isolation given temperature and precipitation in the SE US for *Pa*, temperature and elevation close to the intersection of forests and grasslands for *Ps* and temperate and semiarid prairies for *Lt*, temperature from south to north Texas transitioning between semiarid prairies and Tamaulipan mezquital for *Pe*, and precipitation across the Cochise Filter Barrier for *Ca* and *Ls* (Figures 1 and 3). All of these regions have long been described as biogeographic suture zones at the intersection of major ecoregions in plants and animals (Burbrink, Bernstein, et al., 2022; Burbrink, Gehara, et al., 2021; Myers et al., 2019; Omernik, 1987; Provost et al., 2021; Remington, 1968; Rising, 1983; Soltis et al., 2006; Swenson & Howard, 2005). For those taxa where genetic distance is not entirely defined by spatial distance, this likely indicates unique adaptations to similar biogeographic boundaries. The environmental features examined here may be proxies for other potential biotic and abiotic interactions.

While we identified geographic boundaries and the location of hybrid zones, understanding the dimensionality and potential mosaic nature of these zones is difficult. Inferring genomic clines mitigate this by assessing the change in genotype frequency over admixture (Bailey, 2024; Gompert & Buerkle, 2011; Szymura & Barton, 1986; Figures 1 and 4; Figures S10 and S11). The percentage of loci with significant spatial and genome clines relative to all loci sequenced were not correlated across the taxa considered (*ρ* = −0.448; *p* = 0.26). Genome clines found more loci showing significant selection against introgression, with most located near the center of the area of admixture (Figure 4). These loci indicate that the hybrid zones are likely more complex than can be estimated by inferring spatial clines from phylogeographic data alone (Table 1). This may be perturbed by differences in drift, selection, and hybridization along the lengths of these zones. Many loci appear to be under strong selection within species pairs but there may have also been a role for drift to have shaped these clines, though population sizes appear large for most of these taxa suggesting a stronger role for selection (Figure 5; Felsenstein, 1975; Fitzpatrick, 2013; Polechová & Barton, 2011; Slatkin & Maruyama, 1975).

Similarly, the RDA approach to understanding how species respond to environmental changes via selection generally finds many more significant loci than either genome scan or genome cline methods (Figure 5). Genome scans, genome clines, and DAPC showed more overlap with each other than RDA loci. While there is some overlap among these methods, RDA is likely finding local adaptations (Forester et al., 2018; Jones et al., 2013) along environmental clines within and among lineages but not specifically related to the separation between species. Therefore, this technique may be difficult to use to identify strongly selected loci different between geographically structured populations or lineages. Because RDA requires imputing an average SNP for absent states, the method may be inaccurate with large proportions of missing data (Forester et al., 2018). Similarly, there may be differences between genotype‐environment correlations within each lineage compared to those across both lineages; disentangling this might be an important topic of future research. It is difficult both statistically as well as conceptually, as the present‐day genotype‐environmental association within species may differ from the historical processes between species.

Although species delimitation using genetic data alone has been considered controversial by some researchers (Chambers et al., 2023; Hillis, 2020), we demonstrate how using these data in the broader context of speciation processes removes much of the ambiguity. For all but two taxa, these lineage pairs represent ontologically historically unique and geographically circumscribed individuals (De Queiroz, 2007; Ghiselin, 1974; Hull, 1976; Millstein, 2009). Gene flow occurs between these historically unique lineages and has been considered by some as evidence of either failure to speciate or collapse of species (Chambers et al., 2023; Marshall et al., 2021). In contrast, our results strongly demonstrate that *Pa*, *Ps*, *Pe*, *Lt*, *Ca*, and *Ls* all clearly represent species complexes that are being maintained by selection against 0.29%–0.81% of the loci sampled in the hybrid zone despite historical and contemporary gene flow (Table 1, Figure 5).

Previous research has shown that without selection, extinction via hybridization can occur quickly (Barton & Gale, 1993; Taylor et al., 2006; Vonlanthen et al., 2012; Wolf et al., 2001) and coalescent methods fail to delimit species with as few as one migrant every 10 generations (Zhang et al., 2011). Rather than viewing the hybrid zone as indicating incomplete speciation, we suggest a more robust view conceptualizes the hybrid zone as filtering alleles for key adaptive loci between lineages (Martinsen et al., 2001). In cases of ecological speciation across environmental gradients, a filter zone therefore allows adaptive introgression and prevents incursion of maladaptive alleles and species collapse. These hybrid zones therefore persist over long periods of time, potentially establishing incomplete reproductive isolation as an evolutionary endpoint (Barth et al., 2020; Martinsen et al., 2001; Servedio & Hermisson, 2020).

Furthermore, debates about species delimitation often center on the potential for “oversplitting” of local populations (Hillis, 2020; Sukumaran & Knowles, 2017). We agree that this is possible and suggest range‐wide sampling to ensure prospective species represent historically unique lineages (Burbrink & Ruane, 2021). We show at least two instances (*Pg* and *Pm*) where previously‐established geographic genetic structure does not represent delimitable ecological species. For the species pairs *Pe/Ps* and *Lt/Lg*, some authors have suggested that these represent subspecies given the presence of hybrid zones (Chambers et al., 2023; Marshall et al., 2021). When testing adaptive differentiation across these zones, both species pairs show numerous loci under selection. Furthermore, *Pe/Ps* overlap in the east central Texas plains which represents the transition from the forests of east to the prairies of the west (Omernik, 1987), here showing a steep cline only 36 km wide relative to their 1800 km distributional extent and many loci responding strongly to environmental selection (Figure 1). The hybrid zone for *Lt/Lg* transitioning between temperate and semiarid prairies (Omernik, 1987) represents only a small area of 210 km (6% of the combined ranges) for a complex spanning most of the continent (3200 km) from east to west (Figure S11).

To summarize our perspective: if such historical lineages exist, then they must be species, as there is no way to discriminate between species or subspecies ontologically (Burbrink, Crother, et al., 2022; Cracraft, 1983; Rosen, 1979). Consequently, it does not make sense to demote lineages to subspecies or non‐species based only on the presence of a hybrid zone, particularly given the complexity of clines over long periods of time. Nor does it accurately represent the ecological and evolutionary history of speciation processes.

The obvious question is then: how could divergent lineages remain distinct over time in the face of hybridization unless some form of selection is maintaining their independence? If there is no selection against hybridization across the genome, then it is clearly expected that these lineages would collapse rapidly given the rate of lifetime dispersal and generation length since the Pleistocene (Bailey et al., 2015; Barton & Gale, 1993; Burbrink, Gehara, et al., 2021). As demonstrated, the loci that identify six of these lineage pairs also show evidence for selection against alleles traversing through admixed individuals and thus are maintaining these distinct lineages (Figure S12). We therefore suggest that *Pa*, *Ps*, *Pe*, *Lt*, *Ca*, and *Ls* each represent two species and thus retain those species names in those pairs defined previously (Burbrink, 2002; Burbrink, Bernstein, et al., 2022; Burbrink, Gehara, et al., 2021; Burbrink, Pyron, et al., 2021; Marshall et al., 2021; Myers et al., 2020; Pyron & Burbrink, 2009; Ruane et al., 2014; Smith et al., 1994) or to be described in the case of Ca.

Future work should identify lineages and examine the geographic and environmental context maintaining them to understand the function of loci isolating species as the genomic basis of speciation. This could also be presented in the environmental context of the Pleistocene, responsible for the formation of these lineages, using paleo‐niche modeling. With whole genomes, processes of speciation with gene flow may be teased out from those representing secondary contact and selection (Cruickshank & Hahn, 2014; Irwin et al., 2018). With annotated whole‐genome data, locus numbers, identity, function, recombination, genome architecture and gene ontology responsible for maintaining species boundaries could be better understood (Ashburner et al., 2000; Gene Ontology Consortium et al., 2013; Rautsaw et al., 2021; Wolf & Ellegren, 2017). This will allow us to recognize functional traits being selected for divergence at environmental boundaries across similar taxa or if processes of speciation are idiosyncratic with respect to selected attributes. Nevertheless, it is first necessary to delineate and identify the ranges of species, locate their hybrid zones, and understand the genetic interactions in these areas.

## AUTHOR CONTRIBUTIONS

**Frank T. Burbrink:** Conceptualization (equal); data curation (equal); formal analysis (equal); funding acquisition (equal); investigation (equal); methodology (equal); project administration (equal); resources (lead); validation (lead); visualization (lead); writing – original draft (lead); writing – review and editing (lead). **Edward A. Myers:** Conceptualization (equal); data curation (equal); formal analysis (equal); methodology (equal); writing – review and editing (equal). **R. Alexander Pyron:** Conceptualization (equal); formal analysis (equal); funding acquisition (equal); investigation (equal); methodology (equal); resources (equal); writing – original draft (equal); writing – review and editing (equal).

## CONFLICT OF INTEREST STATEMENT

We declare that there are no competing interests.

## Supporting information


**Figure S1.** (A) Ancestral species coefficients over geography, (B) probabilities of the number of clusters (K) and (C) data layer importance from climate‐based SuperSOMs (alleles, space, and climate) in ‘delim‐som’ (Pyron, 2023) for the Milksnake dataset (Lampropeltis gentilis/triangulum) from Burbrink et al. (2022).

**Figure S2.** (A) Ancestral species coefficients over geography, (B) probabilities of the number of clusters (K) and (C) data layer importance from climate‐based SuperSOMs (alleles, space, and climate) in ‘delim‐som’ (Pyron, 2023) for the Milksnake dataset (Lampropeltis gentilis/triangulum) from Chambers et al. (2023).

**Figure S3.** (A) Ancestral species coefficients over geography, (B) probabilities of the number of clusters (K) and (C) data layer importance from climate‐based SuperSOMs (alleles, space, and climate) in ‘delim‐som’ (Pyron, 2023) for the Cornsnake dataset (Pantherophis emoryi et al./guttatus) from Myers et al. (2020).

**Figure S4.** (A) Ancestral species coefficients over geography, (B) probabilities of the number of clusters (K) and (C) data layer importance from climate‐based SuperSOMs (alleles, space, and climate) in ‘delim‐som’ (Pyron, 2023) for the Cornsnake dataset (Pantherophis emoryi/guttatus/meahllmorum/slowinskii) from Marshall et al. 2021).

**Figure S5.** (A) Ancestral species coefficients over geography, (B) probabilities of the number of clusters (K) and (C) data layer importance from climate‐based SuperSOMs (alleles, space, and climate) in ‘delim‐som’ (Pyron, 2023) for the Ratnake dataset (Pantherophis alleghaniensis/quadrivittatus) from Burbrink et al. (2021).

**Figure S6.** (A) Ancestral species coefficients over geography, (B) probabilities of the number of clusters (K) and (C) data layer importance from climate‐based SuperSOMs (alleles, space, and climate) in ‘delim‐som’ (Pyron, 2023) for the Kingsnake dataset (Lampropeltis californiae/splendida) from Myers et al. (2019).

**Figure S7.** (A) Ancestral species coefficients over geography, (B) probabilities of the number of clusters (K) and (C) data layer importance from climate‐based SuperSOMs (alleles, space, and climate) in ‘delim‐som’ (Pyron, 2023) for the Diamondback Rattlesnake dataset (Crotalus atrox) from Schield et al. (2015).

**Figure S8.** Bivariate scatterplots showing the relationship between ancestry/admixture estimates from TESS3r (described above) versus the species coefficients from climate‐based SuperSOMs (alleles, space, and climate) in ‘delim‐som’ (Pyron, 2023). For the Milk Snakes (Lampropeltis triangulum/gentilis) we overlaid estimates from the two different datasets of Burbrink et al. (2022) in black and Chambers et al. (2023) in gray.

**Figure S9.** Graphs showing estimated cline widths and cline centers for all loci between species‐pair comparisons using HZAR.

**Figure S10.** (A) The location of lineages and interpolated contour clines defining the extent of hybrid zones, (B) loess plot showing individual distance to the cline center against admixture, C) cline estimates from HZAR, and D) density of admixture when combining admixture data from Burbrink et al. (2022) and Chambers et al. (2023) using TESS3r for Lampropeltis triangulum and L. gentilis.

**Figure S11.** The number of loci significantly correlated to changes in indicated environmental variables for each species pairs using redundancy analyses (RDA).

**Figure S12.** Maps showing the location of lineages using only loci that are significant among genome clines, genome scans, and DAPC for all lineage pairs. Values above each graph show the number of loci (and percentage of total loci used here) and Fst values for those reduced loci between geographic lineages.

**Table S1.** References for original data, parameter inputs and results for historical demographic analyses using GADMA. In the results, parameters ending in H, L, and M refer to high, low and medium substitution rates respectively (see text).

**Table S2.** Gadma results.

## Data Availability

All genome‐scale datasets, sample localities, environmental data, and code with worked example are available on FigShare at: https://figshare.com/s/f997bbeb7c465bc411e0.
