# Supplementary figures and images for: Understanding species limits through the formation of phylogeographic lineages
Source: Ecol Evol. 2024 Oct 2;14(10):e70263. doi: 10.1002/ece3.70263 (PMC11446989; doi:10.1002/ece3.70263)

**A)**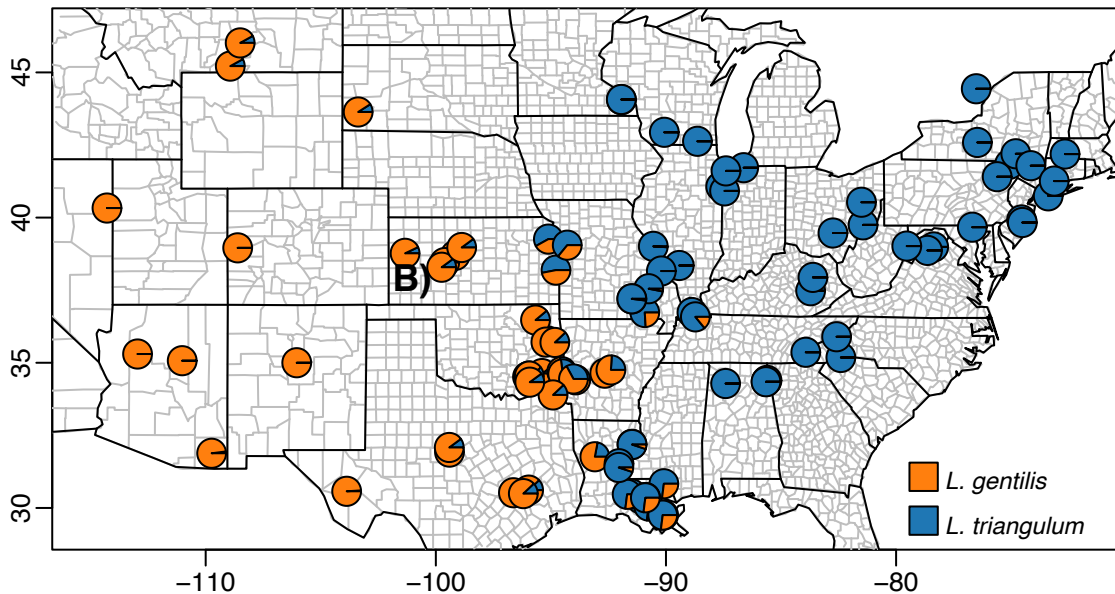**B)**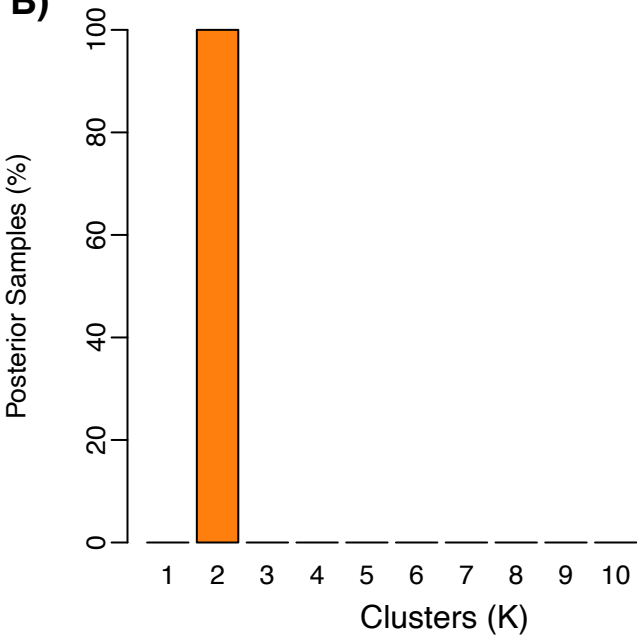**C)**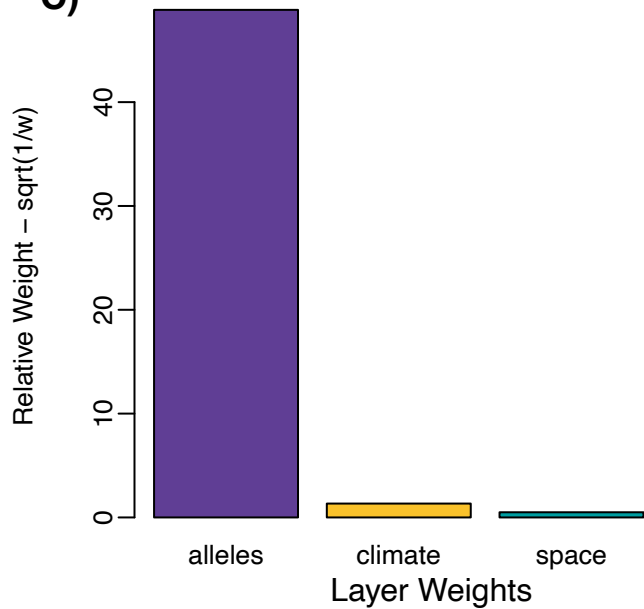

Supplement: Supplementary file 1 — Figure S1. (A) Ancestral species coefficients over geography, (B) probabilities of the number of clusters (K) and (C) data layer importance from climate‐based SuperSOMs (alleles, space, and climate) in ‘delim‐som’ (Pyron, 2023) for the Milksnake dataset (Lampropeltis gentilis/triangulum) from Burbrink et al. (2022). [file ECE3-14-e70263-s005.pdf]

**A)**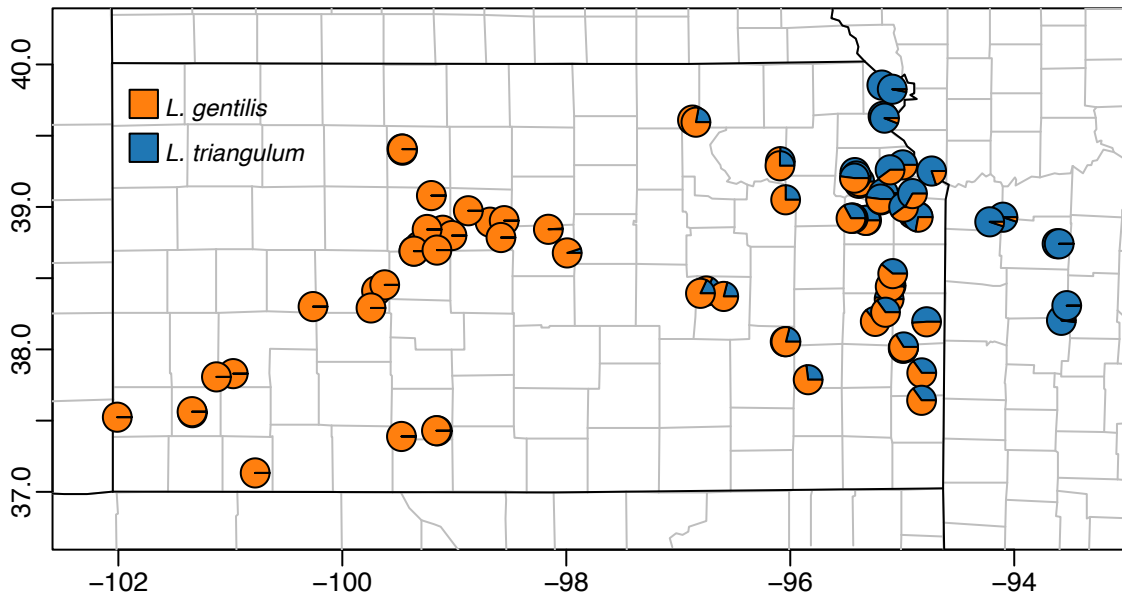**B)**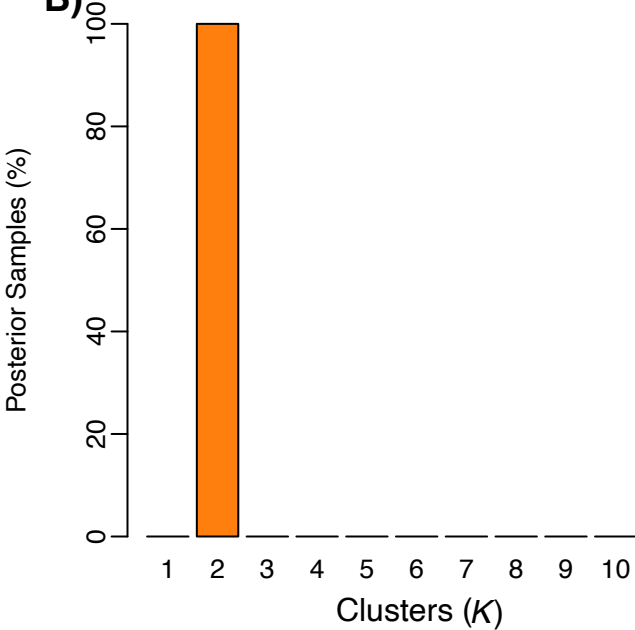**C)**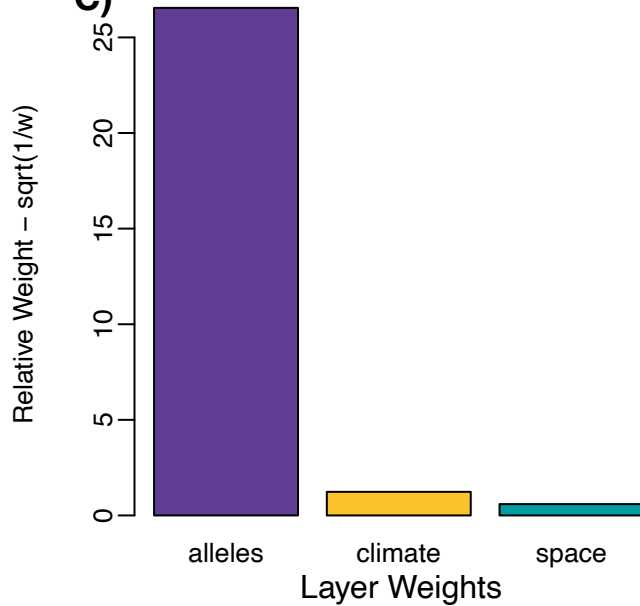

Supplement: Supplementary file 2 — Figure S2. (A) Ancestral species coefficients over geography, (B) probabilities of the number of clusters (K) and (C) data layer importance from climate‐based SuperSOMs (alleles, space, and climate) in ‘delim‐som’ (Pyron, 2023) for the Milksnake dataset (Lampropeltis gentilis/triangulum) from Chambers et al. (2023). [file ECE3-14-e70263-s006.pdf]

**A)**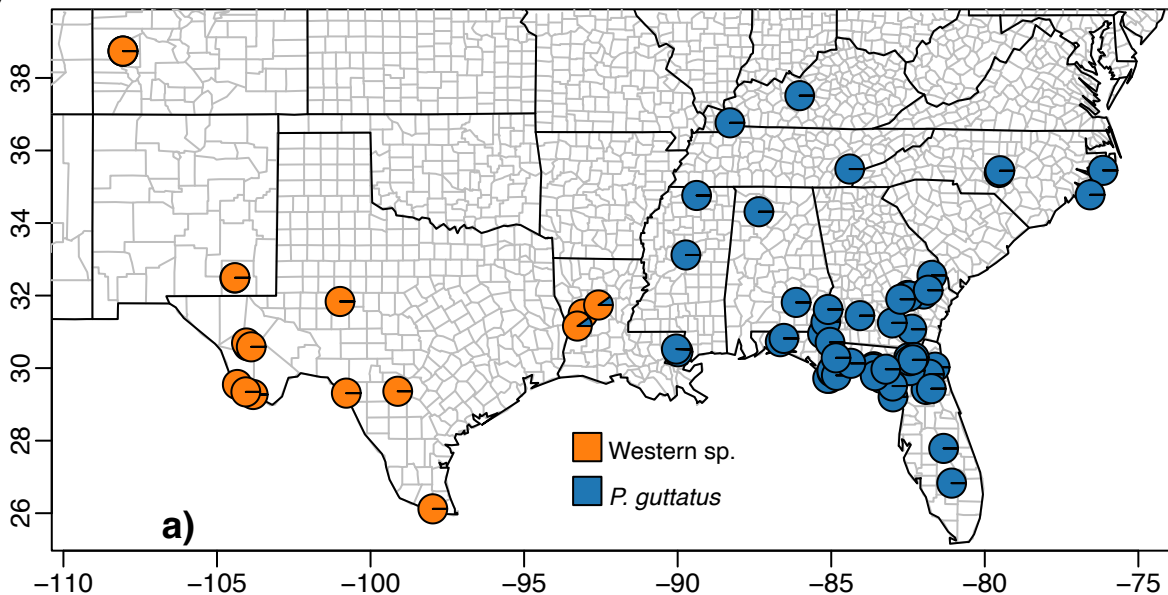**B)**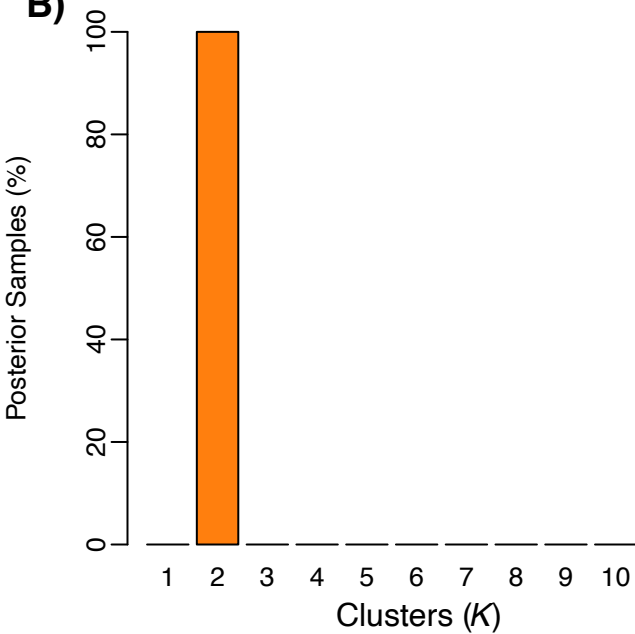**C)**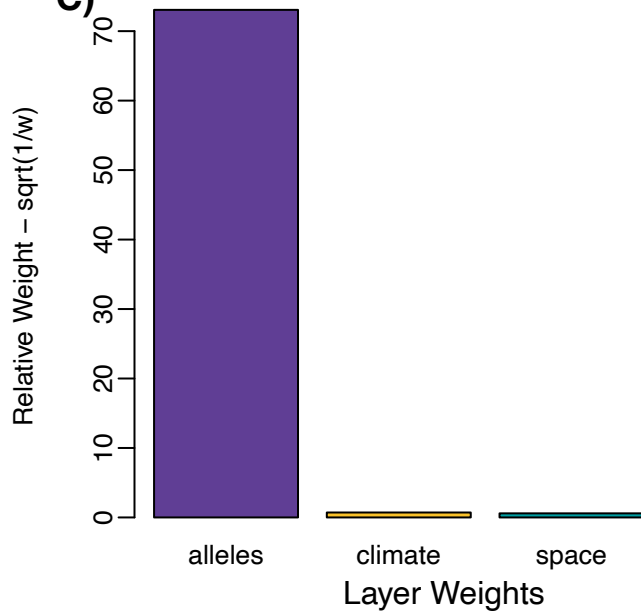

Supplement: Supplementary file 3 — Figure S3. (A) Ancestral species coefficients over geography, (B) probabilities of the number of clusters (K) and (C) data layer importance from climate‐based SuperSOMs (alleles, space, and climate) in ‘delim‐som’ (Pyron, 2023) for the Cornsnake dataset (Pantherophis emoryi et al./guttatus) from Myers et al. (2020). [file ECE3-14-e70263-s001.pdf]

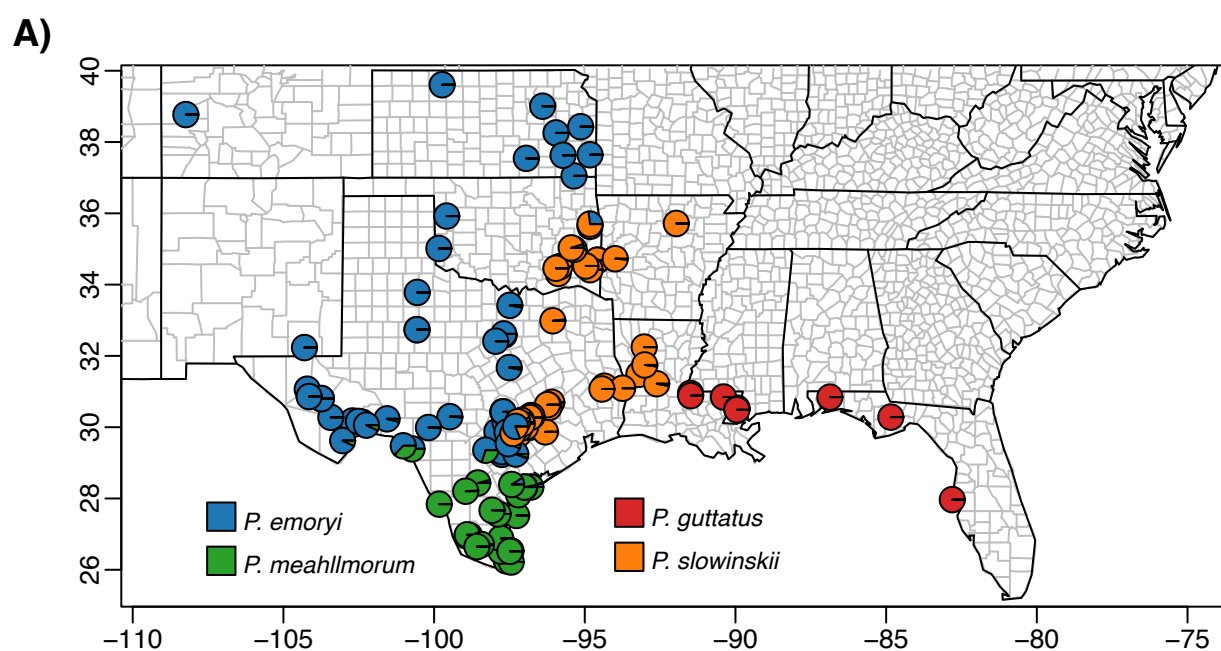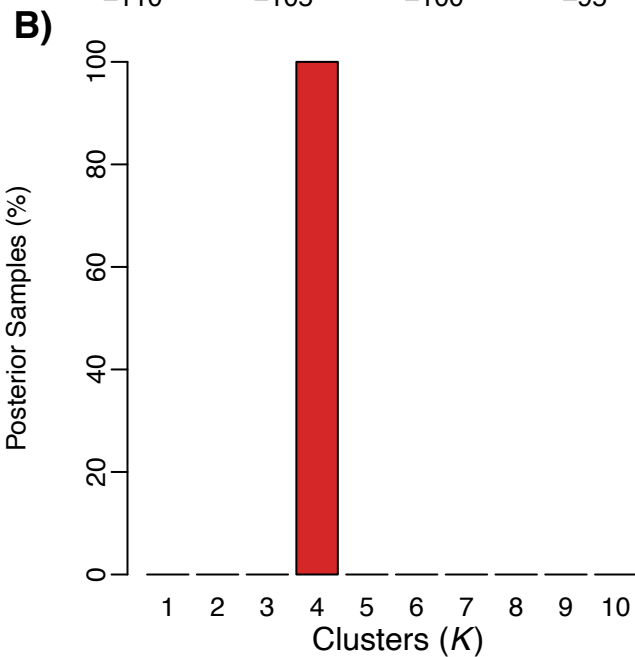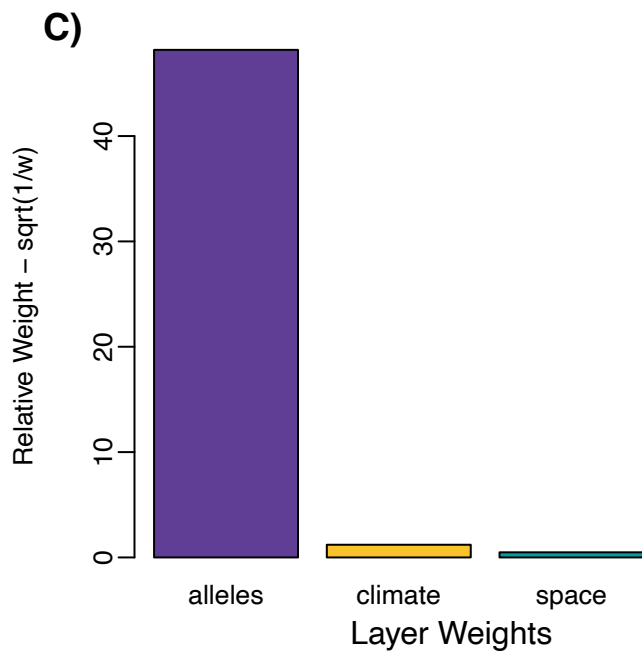

Supplement: Supplementary file 4 — Figure S4. (A) Ancestral species coefficients over geography, (B) probabilities of the number of clusters (K) and (C) data layer importance from climate‐based SuperSOMs (alleles, space, and climate) in ‘delim‐som’ (Pyron, 2023) for the Cornsnake dataset (Pantherophis emoryi/guttatus/meahllmorum/slowinskii) from Marshall et al. 2021). [file ECE3-14-e70263-s011.pdf]

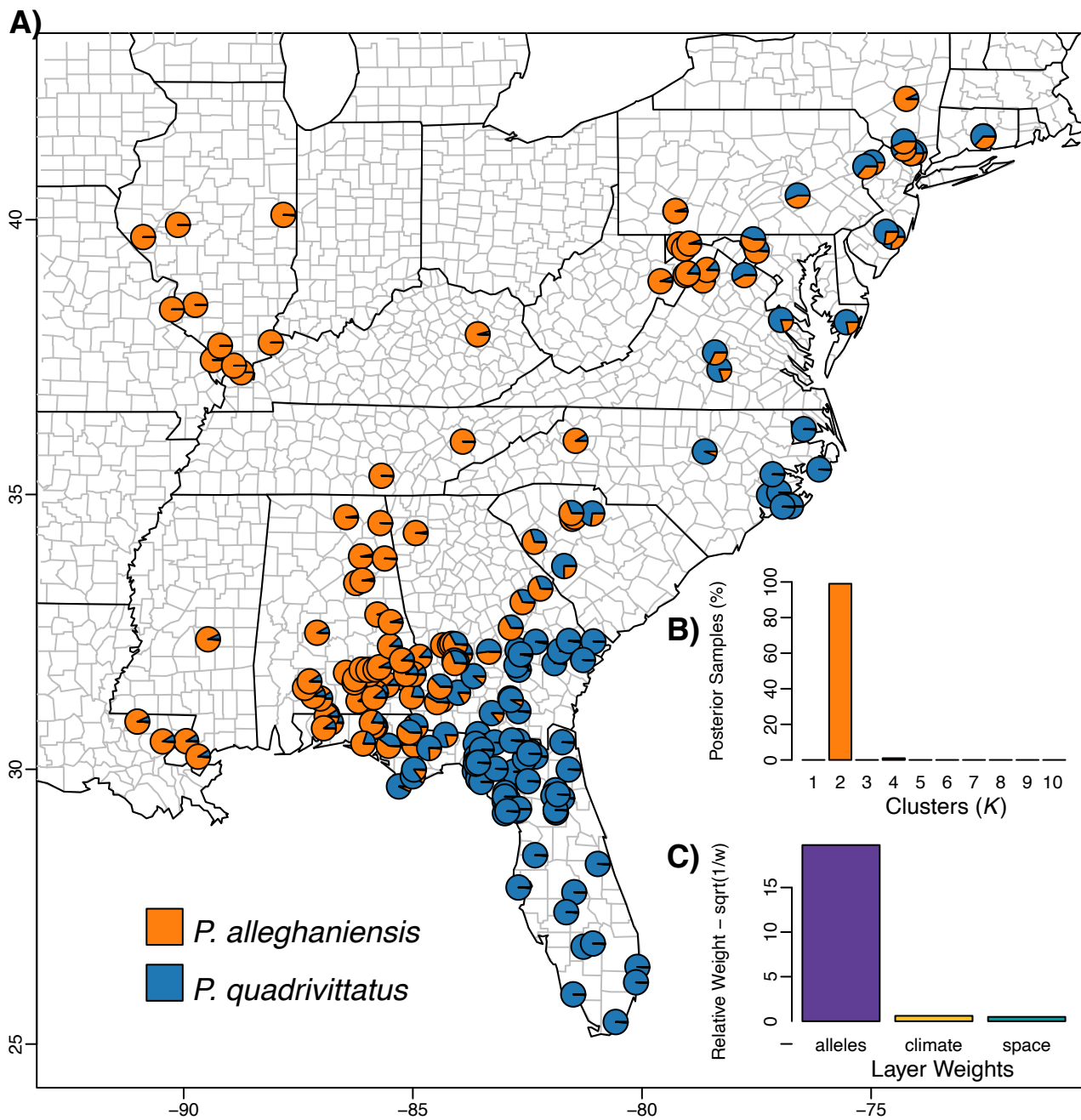

Supplement: Supplementary file 5 — Figure S5. (A) Ancestral species coefficients over geography, (B) probabilities of the number of clusters (K) and (C) data layer importance from climate‐based SuperSOMs (alleles, space, and climate) in ‘delim‐som’ (Pyron, 2023) for the Ratnake dataset (Pantherophis alleghaniensis/quadrivittatus) from Burbrink et al. (2021). [file ECE3-14-e70263-s007.pdf]

**A)**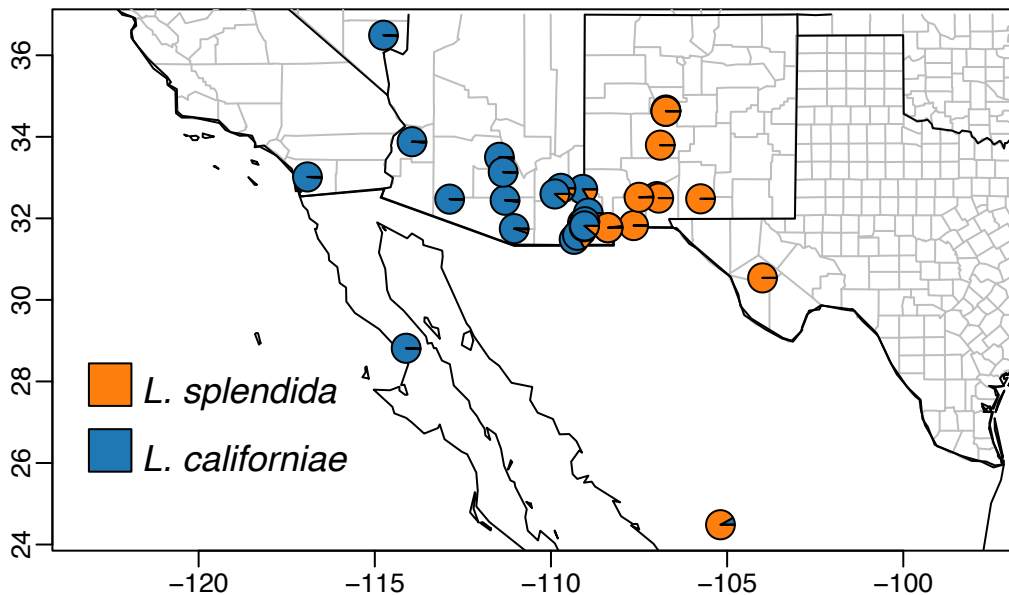**B)**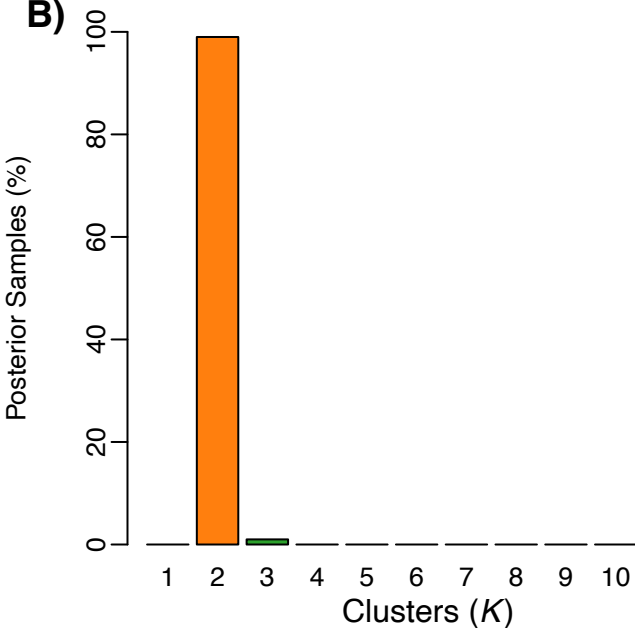**C)**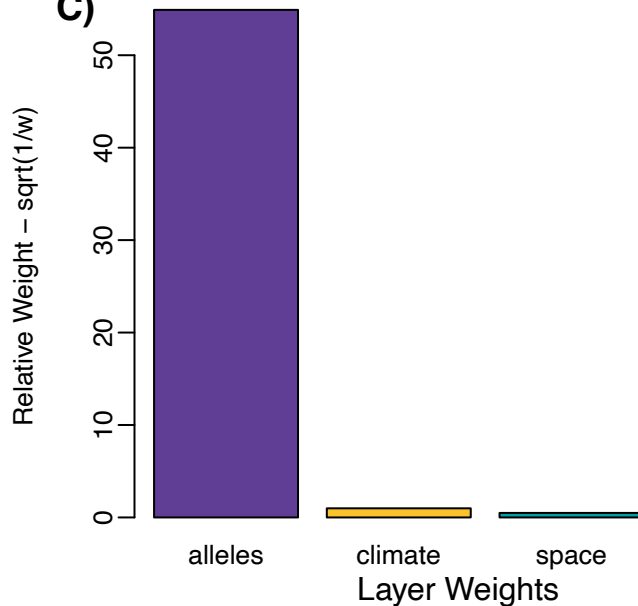

Supplement: Supplementary file 6 — Figure S6. (A) Ancestral species coefficients over geography, (B) probabilities of the number of clusters (K) and (C) data layer importance from climate‐based SuperSOMs (alleles, space, and climate) in ‘delim‐som’ (Pyron, 2023) for the Kingsnake dataset (Lampropeltis californiae/splendida) from Myers et al. (2019). [file ECE3-14-e70263-s013.pdf]

**A)**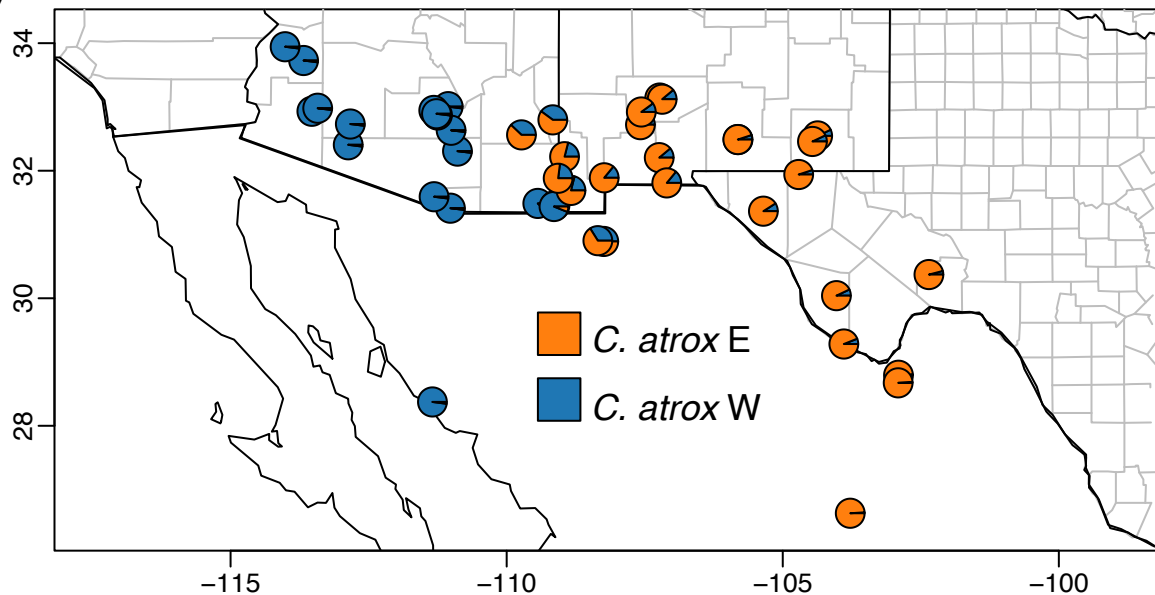**B)**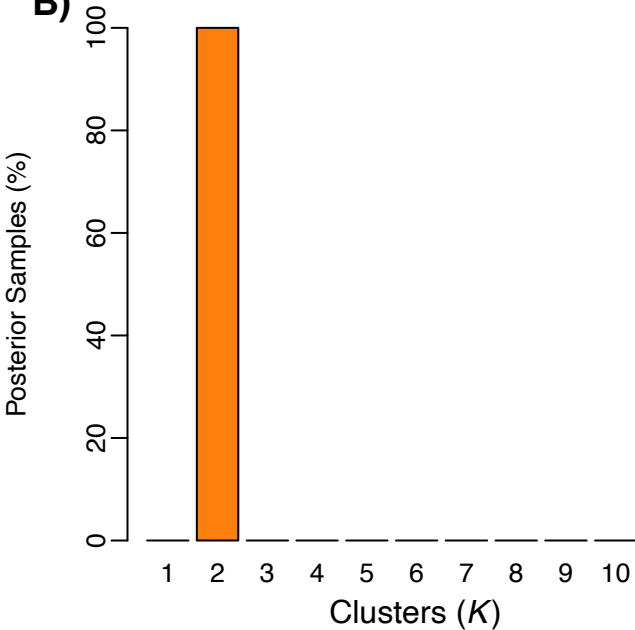**C)**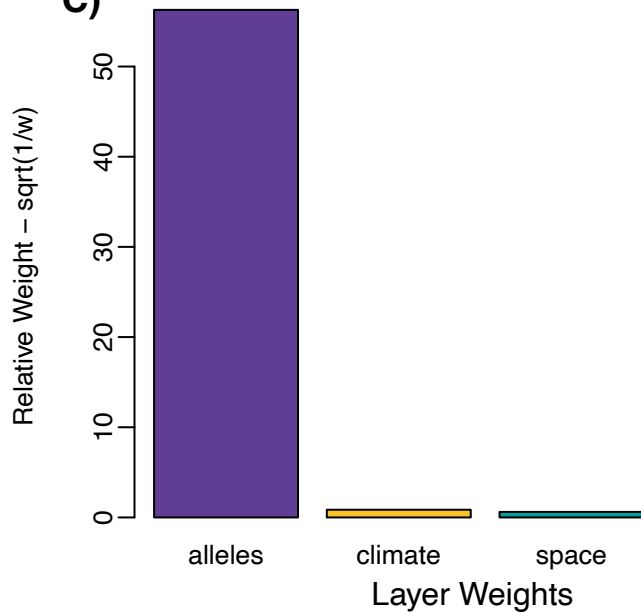

Supplement: Supplementary file 7 — Figure S7. (A) Ancestral species coefficients over geography, (B) probabilities of the number of clusters (K) and (C) data layer importance from climate‐based SuperSOMs (alleles, space, and climate) in ‘delim‐som’ (Pyron, 2023) for the Diamondback Rattlesnake dataset (Crotalus atrox) from Schield et al. (2015). [file ECE3-14-e70263-s009.pdf]

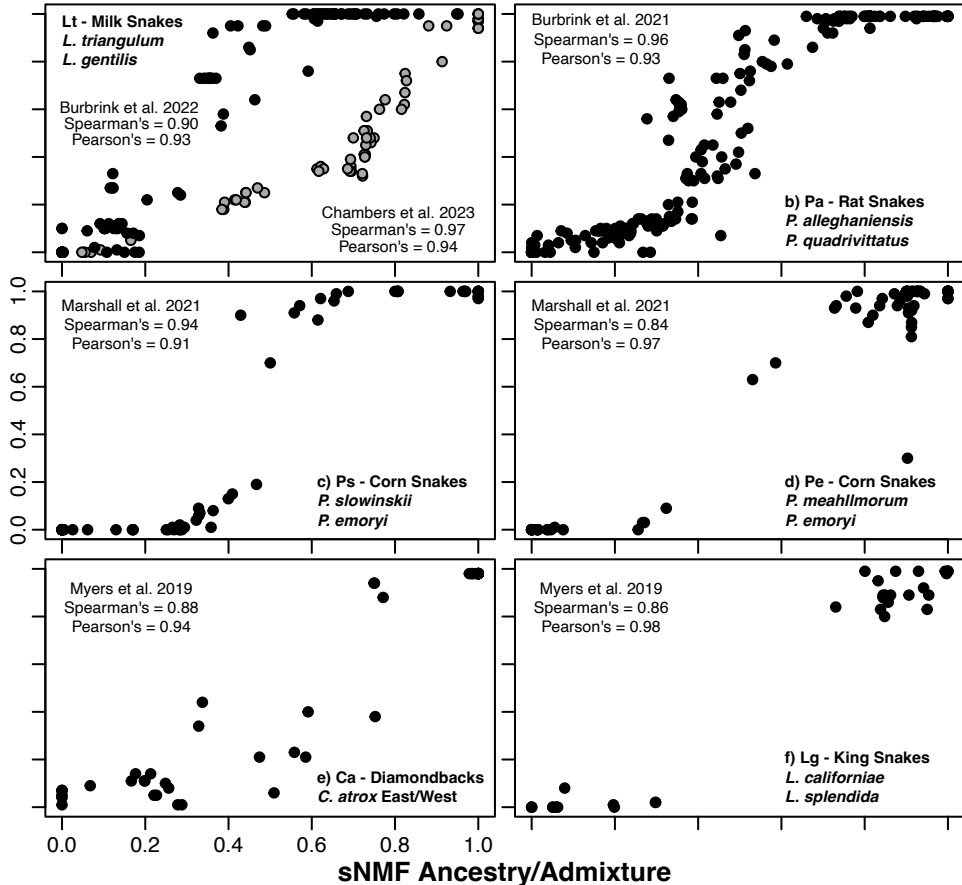

Supplement: Supplementary file 8 — Figure S8. Bivariate scatterplots showing the relationship between ancestry/admixture estimates from TESS3r (described above) versus the species coefficients from climate‐based SuperSOMs (alleles, space, and climate) in ‘delim‐som’ (Pyron, 2023). For the Milk Snakes (Lampropeltis triangulum/gentilis) we overlaid estimates from the two different datasets of Burbrink et al. (2022) in black and Chambers et al. (2023) in gray. [file ECE3-14-e70263-s003.pdf]
