# Supplementary material for: Understanding species limits through the formation of phylogeographic lineages
Source: Ecol Evol. 2024 Oct 2;14(10):e70263. doi: 10.1002/ece3.70263 (PMC11446989; doi:10.1002/ece3.70263)
Supplement: Supplementary file 9 — Figure S9. Graphs showing estimated cline widths and cline centers for all loci between species‐pair comparisons using HZAR. [file ECE3-14-e70263-s004.pdf]

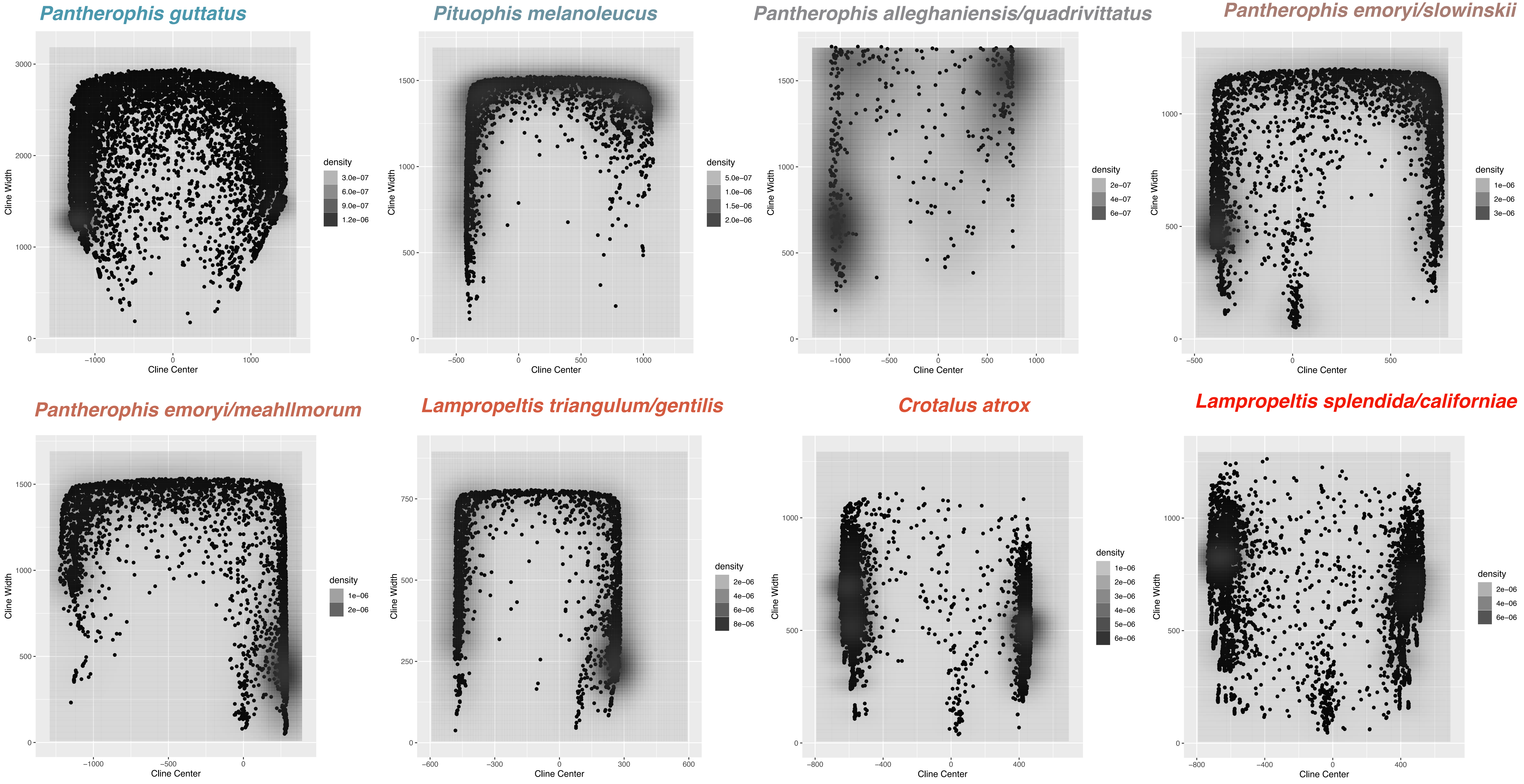
Pantherophis guttatus
Pituophis melanoleucus
Pantherophis alleghaniensis/quadrivittatus
Pantherophis emoryi/slowinskii
Pantherophis emoryi/meahllmorum
Lampropeltis triangulum/gentilis
Crotalus atrox
Lampropeltis splendida/californiae
Cline Width
Cline Center
density
3.0e−07
6.0e−07
9.0e−07
1.2e−06
5.0e−07
1.0e−06
1.5e−06
2.0e−06
2e−07
4e−07
6e−07
1e−06
2e−06
3e−06
4e−06
5e−06
6e−06
8e−06
