# Supplementary material for: Understanding species limits through the formation of phylogeographic lineages
Source: Ecol Evol. 2024 Oct 2;14(10):e70263. doi: 10.1002/ece3.70263 (PMC11446989; doi:10.1002/ece3.70263)
Supplement: Supplementary file 10 — Figure S10. (A) The location of lineages and interpolated contour clines defining the extent of hybrid zones, (B) loess plot showing individual distance to the cline center against admixture, C) cline estimates from HZAR, and D) density of admixture when combining admixture data from Burbrink et al. (2022) and Chambers et al. (2023) using TESS3r for Lampropeltis triangulum and L. gentilis. [file ECE3-14-e70263-s014.pdf]

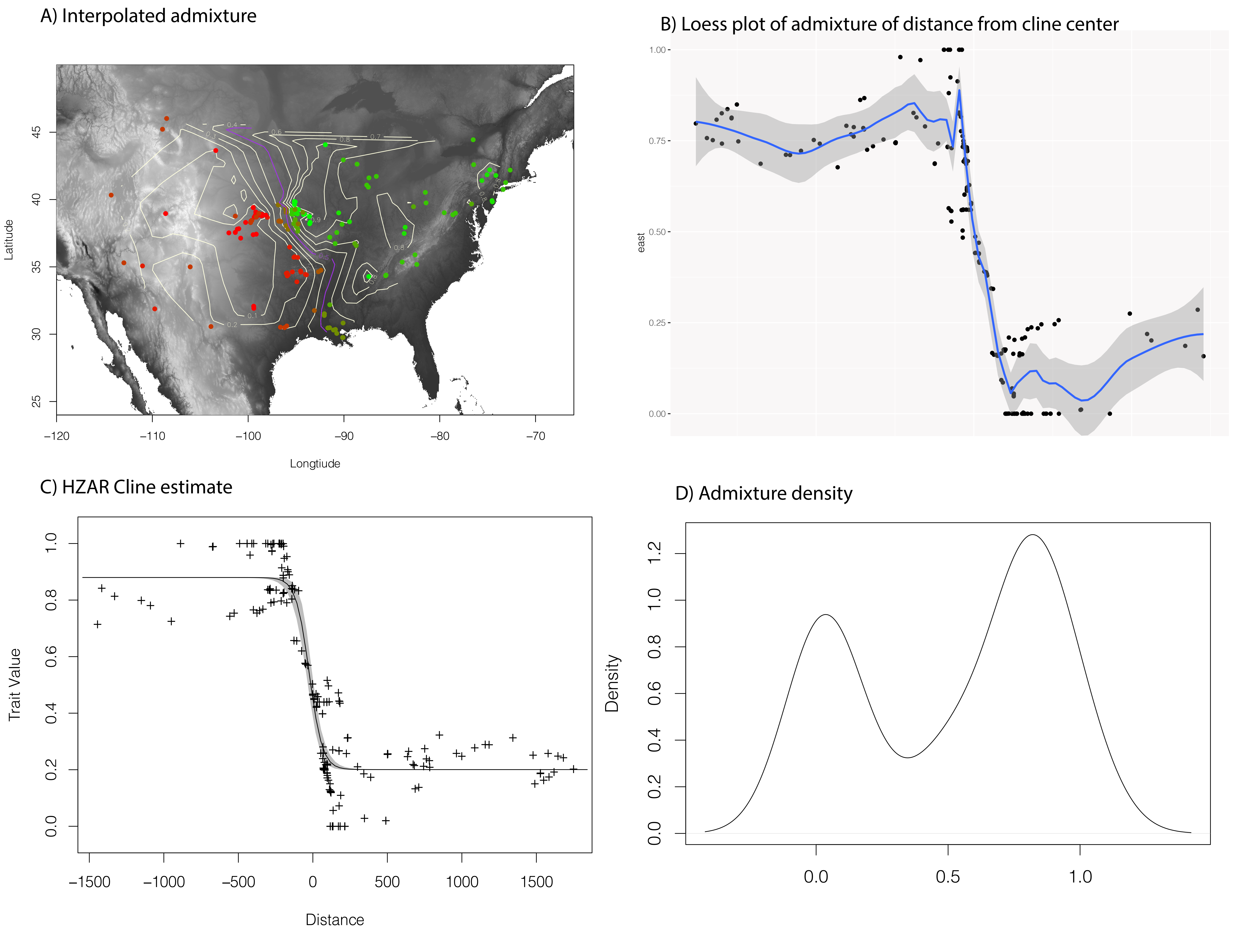

A) Interpolated admixture
Latitude
Longtiude
−120
−110
−100
−90
−80
−70
25
30
35
40
45
B) Loess plot of admixture of distance from cline center
east
0.00
0.25
0.50
0.75
1.00
C) HZAR Cline estimate
Trait Value
Distance
−1500
−1000
−500
0
500
1000
1500
0.0
0.2
0.4
0.6
0.8
1.0
D) Admixture density
Density
0.0
0.5
1.0
0.0
0.2
0.4
0.6
0.8
1.0
1.2
