# Supplementary material for: Understanding species limits through the formation of phylogeographic lineages
Source: Ecol Evol. 2024 Oct 2;14(10):e70263. doi: 10.1002/ece3.70263 (PMC11446989; doi:10.1002/ece3.70263)
Supplement: Supplementary file 11 — Figure S11. The number of loci significantly correlated to changes in indicated environmental variables for each species pairs using redundancy analyses (RDA). [file ECE3-14-e70263-s012.pdf]

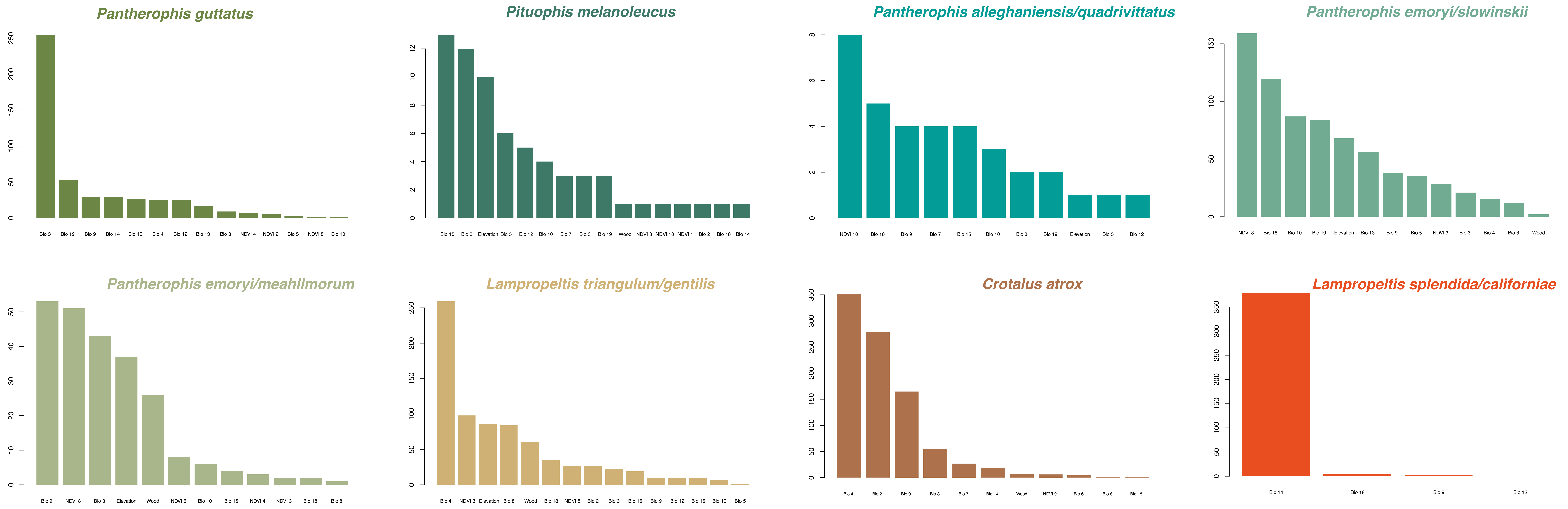

Pantherophis guttatus
0 50 100 150 200 250
Bio 3 Bio 19 Bio 9 Bio 14 Bio 15 Bio 4 Bio 12 Bio 13 Bio 8 NDVI 4 NDVI 2 Bio 5 NDVI 8 Bio 10
Pituophis melanoleucus
0 2 4 6 8 10 12
Bio 15 Bio 8 Elevation Bio 5 Bio 12 Bio 10 Bio 7 Bio 3 Bio 19 Wood NDVI 8 NDVI 10 NDVI 1 Bio 2 Bio 18 Bio 14
Pantherophis alleghaniensis/quadrivittatus
0 2 4 6 8
NDVI 10 Bio 18 Bio 9 Bio 7 Bio 15 Bio 10 Bio 3 Bio 19 Elevation Bio 5 Bio 12
Pantherophis emoryi/slowinskii
0 50 100 150
NDVI 8 Bio 18 Bio 10 Bio 19 Elevation Bio 13 Bio 9 Bio 5 NDVI 3 Bio 3 Bio 4 Bio 8 Wood
Pantherophis emoryi/meahllmorum
0 10 20 30 40 50
Bio 9 NDVI 8 Bio 3 Elevation Wood NDVI 6 Bio 10 Bio 15 NDVI 4 NDVI 3 Bio 18 Bio 8
Lampropeltis triangulum/gentilis
0 50 100 150 200 250
Bio 4 NDVI 3 Elevation Bio 8 Wood Bio 18 NDVI 8 Bio 2 Bio 3 Bio 16 Bio 9 Bio 12 Bio 15 Bio 10 Bio 5
Crotalus atrox
0 50 100 150 200 250 300 350
Bio 4 Bio 2 Bio 9 Bio 3 Bio 7 Bio 14 Wood NDVI 9 Bio 6 Bio 8 Bio 15
Lampropeltis splendida/californiae
0 50 100 150 200 250 300 350
Bio 14 Bio 18 Bio 9 Bio 12
