# Supplementary material for: Understanding species limits through the formation of phylogeographic lineages
Source: Ecol Evol. 2024 Oct 2;14(10):e70263. doi: 10.1002/ece3.70263 (PMC11446989; doi:10.1002/ece3.70263)
Supplement: Supplementary file 12 — Figure S12. Maps showing the location of lineages using only loci that are significant among genome clines, genome scans, and DAPC for all lineage pairs. Values above each graph show the number of loci (and percentage of total loci used here) and Fst values for those reduced loci between geographic lineages. [file ECE3-14-e70263-s010.pdf]

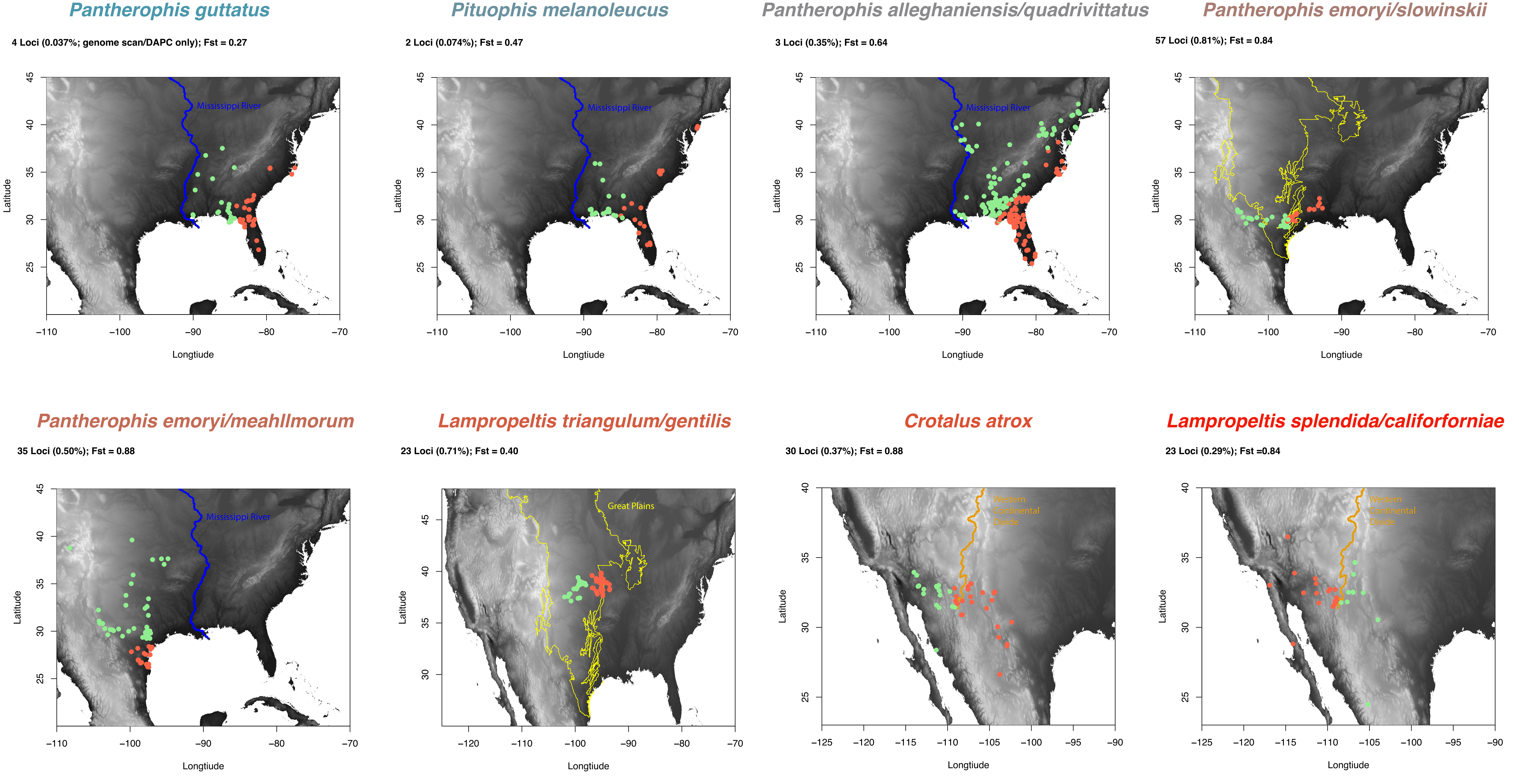

Pantherophis guttatus
4 Loci (0.037%; genome scan/DAPC only); Fst = 0.27
Pituophis melanoleucus
2 Loci (0.074%); Fst = 0.47
Pantherophis alleghaniensis/quadrivittatus
3 Loci (0.35%); Fst = 0.64
Pantherophis emoryi/slowinskii
57 Loci (0.81%); Fst = 0.84
Mississippi River
Latitude
Longtiude
Pantherophis emoryi/meahllmorum
35 Loci (0.50%); Fst = 0.88
Lampropeltis triangulum/gentilis
23 Loci (0.71%); Fst = 0.40
Crotalus atrox
30 Loci (0.37%); Fst = 0.88
Lampropeltis splendida/califorforniae
23 Loci (0.29%); Fst =0.84
Great Plains
Western
Continental
Divide
