# Supplementary material for: Understanding species limits through the formation of phylogeographic lineages
Source: Ecol Evol. 2024 Oct 2;14(10):e70263. doi: 10.1002/ece3.70263 (PMC11446989; doi:10.1002/ece3.70263)
Supplement: Supplementary file 13 — Table S1. References for original data, parameter inputs and results for historical demographic analyses using GADMA. In the results, parameters ending in H, L, and M refer to high, low and medium substitution rates respectively (see text). [file ECE3-14-e70263-s002.docx]

Table S1. References for original data, parameter inputs and results for historical demographic analyses using GADMA. In the results, parameters ending in H, L, and M refer to high, low and medium substitution rates respectively (see text).

| Clade | *Pantherophis guttatus* eastern | *Pantherophis*  *Alleghaniensis/qudrivittatus* | *Lampropetlis Triangulum / gentilis* | *Lampropetlis Triangulum / gentilis* | *Crotalus atrox* Cochise | *Lampropeltis splendida/californiae* |
| --- | --- | --- | --- | --- | --- | --- |
| Reference | Myers et al. 2020 | Burbrink et al. 2021 | Chambers et al. 2023 | Burbrink et al. 2022 | Myers et al. 2019 | Myers et al. 2019 |
| GenomeData | 371138 | 866121 | 840318 | 237665 | 593481 | 603195 |
| Total SNPs | 19545 | 34854 | 50852 | 8622 | 7952 | 7879 |
| SFS Projections | 16,18 | 19,17 | 14,16 | 14,14 | 14,14 | 8,8 |
| Generation Time | 2.5 | 3.5 | 2.5 | 2.5 | 3.5 | 2.5 |
|  |  |  |  |  |  |  |
| Results: | Ancestral Ne |  |  |  |  |  |
| NancH | 93042.27 | 15059.08 | 236243.70 | 55458.92 | 4099.07 | 5440.35 |
| NancL | 454939.04 | 75305.37 | 1289198.50 | 288865.30 | 20537.59 | 27191.81 |
| NancM | 149126.49 | 25097.06 | 397164.55 | 97001.13 | 6810.80 | 9060.41 |
|  |  |  |  |  |  |  |
|  | Divergence time in years |  |  |  |  |  |
| TauH | 879873.74 | 96680.61 | 394195.97 | 517053.43 | 143467.48 | 136008.66 |
| TauL | 5058898.22 | 483352.57 | 1808396.68 | 2455071.49 | 718815.72 | 679795.18 |
| TauM | 1798549.68 | 161136.88 | 642863.14 | 806850.47 | 238377.88 | 226510.29 |
|  |  |  |  |  |  |  |
|  | Initial Ne of the ancestral lineage to Na |  |  |  |  |  |
| N0H | 19426.16 | 4730.06 | 236007.46 | 55255.42 | 158.88 | 1078.99 |
| N0L | 61252.38 | 23677.46 | 1287909.30 | 287163.76 | 768.40 | 5194.09 |
| N0M | 17482.16 | 7894.67 | 396767.39 | 96421.90 | 272.57 | 1742.92 |
|  |  |  |  |  |  |  |
|  | Initial Ne of the ancestral lineage to Nb |  |  |  |  |  |
| N1H | 73616.11 | 10329.02 | 236.24 | 203.51 | 3940.19 | 4361.36 |
| N1L | 393686.66 | 51627.91 | 1289.20 | 1701.53 | 19769.19 | 21997.71 |
| N1M | 131644.33 | 17202.39 | 397.16 | 579.23 | 6538.22 | 7317.50 |
|  |  |  |  |  |  |  |
|  | Ne of the first species in the present day |  |  |  |  |  |
| NaH | 46154.57 | 658762.10 | 85841.94 | 205593.25 | 409907.08 | 475307.40 |
| NaL | 228661.25 | 3295012.14 | 373720.63 | 1032243.36 | 2053759.20 | 2433868.46 |
| NaM | 75390.43 | 1098297.67 | 143120.80 | 347852.54 | 681079.67 | 813147.87 |
|  |  |  |  |  |  |  |
|  | Ne of the second species in the present day |  |  |  |  |  |
| NbH | 129448.52 | 978411.12 | 141913.49 | 210563.38 | 203490.66 | 544034.64 |
| NbL | 646876.28 | 4893225.76 | 716379.50 | 1026421.76 | 1011596.36 | 2719180.73 |
| NbM | 216131.42 | 1630029.96 | 234365.54 | 344054.45 | 343594.56 | 906041.17 |
|  |  |  |  |  |  |  |
|  | Migration rate in individuals per generation from 2 into 1 |  |  |  |  |  |
| m12H | 20.00 | 1.43 | 16.57 | 0.36 | 0.10 | 0.03 |
| m12L | 20.00 | 1.42 | 20.00 | 0.37 | 0.10 | 0.03 |
| m12M | 20.00 | 1.42 | 16.93 | 0.37 | 0.10 | 0.04 |
|  |  |  |  |  |  |  |
|  | Migration rate in individuals per generation from 1 into 2 |  |  |  |  |  |
| m21H | 7.37 | 1.45 | 8.93 | 1.85 | 0.03 | 0.08 |
| m21L | 7.05 | 1.45 | 9.15 | 1.91 | 0.03 | 0.08 |
| m21M | 6.81 | 1.45 | 9.02 | 1.92 | 0.03 | 0.08 |
|  |  |  |  |  |  |  |
|  | Theta |  |  |  |  |  |
| thetaH | 1381.26 | 521.72 | 7940.79 | 527.23 | 97.31 | 131.26 |
| thetaL | 1350.76 | 521.79 | 8666.69 | 549.23 | 97.51 | 131.22 |
| thetaM | 1328.32 | 521.69 | 8009.87 | 553.29 | 97.01 | 131.16 |
|  |  |  |  |  |  |  |

| Clade | *Pantherophis slowinskii/emoryi/meahllmorus* |
| --- | --- |
| Reference | Marshall et al. 2021 |
| GenomeData | 1324661 |
| Total SNPs | 71504 |
| SFS Projections | 20,25,16 |
| Generation Time | 2.5 |
|  |  |
| Results: | Ancestral Ne |
| NancH | 90879.13 |
| NancL | 703704.18 |
| NancM | 231106.12 |
|  |  |
|  | Time of the earliest split in years |
| TauH | 754578.19 |
| TauL | 3755001.09 |
| TauM | 813154.64 |
|  |  |
|  | Initial Ne of the first ancestral lineage from the earliest split |
| NaH | 2011.4 |
| NaL | 8461.26 |
| NaM | 6669.53 |
|  |  |
|  | Initial Ne of the second ancestral lineage from the earliest split |
| NbH | 5620844.25 |
| NbL | 28720472.34 |
| NbM | 1313227.36 |
|  |  |
|  | Migration rate between the ancestral lineages |
| m12H | 0 |
| m12L | 16.28 |
| m12M | 0 |
|  |  |
|  | Migration rate between the ancestral lineages |
| m21H | 0 |
| m21L | 4.18 |
| m21M | 0.08 |
|  |  |
|  | Ending Ne of the first ancestral lineage from the earliest split |
| Nb1H | 9625.467953 |
| Nb1L | 56347.77022 |
| Nb1M | 19166.19936 |
|  |  |
|  | Ending Ne of the second ancestral lineage from the earliest split |
| Nb2H | 8518.127361 |
| Nb2L | 31625.10594 |
| Nb2M | 9917.321167 |
|  | time of the most recent split in years |
| Tau2H | 130637.81 |
| Tau2L | 579989.15 |
| Tau2M | 168348.6 |
|  |  |
|  | Ne of slowinskii |
| N1H | 97765.57 |
| N1L | 501198.64 |
| N1M | 158358.96 |
|  |  |
|  | Ne of emoryi |
| N2H | 135136.14 |
| N2L | 704219.79 |
| N2M | 241525.61 |
|  |  |
|  | Ne of meahllmorum |
| N3H | 55170.37 |
| N3L | 354748.32 |
| N3M | 130241.56 |
|  |  |
|  | Migration rates between extant species in migrants per generation |
| m2_12H | 7.13 |
| m2_12L | 12.77 |
| m2_12M | 11.17 |
|  |  |
|  | Migration rates between extant species in migrants per generation |
| m2_13H | 0.64 |
| m2_13L | 0.68 |
| m2_13M | 0.69 |
|  |  |
|  | Migration rates between extant species in migrants per generation |
| m2_21H | 3.66 |
| m2_21L | 5.54 |
| m2_21M | 5.38 |
|  |  |
|  | Migration rates between extant species in migrants per generation |
| m2_23H | 4.39 |
| m2_23L | 6.61 |
| m2_23M | 6.01 |
|  |  |
|  | Migration rates between extant species in migrants per generation |
| m2_31H | 0.48 |
| m2_31L | 0.58 |
| m2_31M | 0.51 |
|  |  |
|  | Migration rates between extant species in migrants per generation |
| m2_32H | 5.56 |
| m2_32L | 8.92 |
| m2_32M | 8.15 |
|  |  |
|  | Theta |
| thetaH | 5180.55 |
| thetaL | 8159.37 |
| thetaM | 7347.29 |
