# Supplementary material for: Understanding species limits through the formation of phylogeographic lineages
Source: Ecol Evol. 2024 Oct 2;14(10):e70263. doi: 10.1002/ece3.70263 (PMC11446989; doi:10.1002/ece3.70263)
Supplement: Supplementary file 14 — Table S2. Gadma results. [file ECE3-14-e70263-s008.docx]

Table S2. Results from isolation and migration models using GADMA showing taxon comparisons, datasets, age of divergence, migration rates (individuals/generation) and expansion ratio (contemporary Ne/ancestral Ne).

| Taxon | Dataset | Age | Migration (E->W) | Migration (W->E) | Expansion (E) | Expansion (W) |
| --- | --- | --- | --- | --- | --- | --- |
| **Pantherophis guttatus (eastern lineages)** | Myers et al. 2020 | 1798.5 (879.9-5058.9) | 6.81 (7.05-7.37) | 20 (20-20) | 3.6 | 1.7 |
| **Pantherophis alleghaniensis / P. quadrivittatus** | Burbrink et al. 2021 | 161.1 (96.7-483.4) | 1.45 (1.45-1.45) | 1.42 (1.42-1.43) | 94.8 | 139.2 |
| **Lampropeltis gentilis / L. triangulum** | Burbrink et al. 2022 | 806.9 (517.1-2455.1) | 0.37 (0.36-0.37) | 1.92 (1.85-1.91) | 636.4 | 3.6 |
| **Lampropeltis gentilis / L. triangulum** | Chambers et al. 2023 | 642.9 (394.2-1808.4) | 9.02 (8.93-9.15) | 16.93 (16.57-20) | 0.3 | 568.3 |
| **Lampropeltis splendida / L. californiae** | Myers et al. 2019 | 226.5 (136-679.8) | 0.08 (0.08-0.08) | 0.04 (0.03-0.03) | 464.4 | 123.8 |
| **Crotalus atrox (Cochise lineages)** | Myers et al. 2019 | 238.4 (143.5-718.8) | 0.1 (0.1-0.1) | 0.03 (0.03-0.03) | 51.5 | 2620.9 |

| Pantherophis slowinskii / P. emoryi/ P. meahllmorum | Marshall et al. 2021 |  |  |  |  |  |
| --- | --- | --- | --- | --- | --- | --- |
| **Lineage A** | **Lineage B** | **Age** | **Migration (A->B)** | **Migration (B->A)** | **Expansion** | **Expansion** |
| P. slowinskii | *P. emoryi* | 813.2 (754.6-3755) | 5.38 (3.66-5.54) | 11.17 (7.13-12.77) | 12.7 | 44.2 |
| P. slowinskii | *P. meahllmorum* | 813.2 (754.6-3755) | 0.51 (0.48-0.58) | 0.69 (0.64-0.68) | 10.8 | - |
| P. emoryi | *P. meahllmorum* | 168.35 (130.64-579.99) | 8.15 (5.56-8.92) | 6.01 (4.39-6.61) | - | - |
|  |  |  |  |  |  |  |

**Supplementary Figures**

Figure S1. (A) Ancestral species coefficients over geography, (B) probabilities of the number of clusters (K) and (C) data layer importance from climate-based SuperSOMs (alleles, space, and climate) in ‘delim-som’ (Pyron 2023) for the Milksnake dataset (*Lampropeltis gentilis/triangulum*) from Burbrink et al. (2022).

Figure S2. (A) Ancestral species coefficients over geography, (B) probabilities of the number of clusters (K) and (C) data layer importance from climate-based SuperSOMs (alleles, space, and climate) in ‘delim-som’ (Pyron 2023) for the Milksnake dataset (*Lampropeltis gentilis/triangulum*) from Chambers et al. (2023).

Figure S3. (A) Ancestral species coefficients over geography, (B) probabilities of the number of clusters (K) and (C) data layer importance from climate-based SuperSOMs (alleles, space, and climate) in ‘delim-som’ (Pyron 2023) for the Cornsnake dataset (*Pantherophis emory*i et al./ *guttatus*) from Myers et al. (2020).

Figure S4. (A) Ancestral species coefficients over geography, (B) probabilities of the number of clusters (K) and (C) data layer importance from climate-based SuperSOMs (alleles, space, and climate) in ‘delim-som’ (Pyron 2023) for the Cornsnake dataset (*Pantherophis emoryi/guttatus/meahllmorum/slowinskii*) from Marshall et al. 2021).

Figure S5. (A) Ancestral species coefficients over geography, (B) probabilities of the number of clusters (K) and (C) data layer importance from climate-based SuperSOMs (alleles, space, and climate) in ‘delim-som’ (Pyron 2023) for the Ratnake dataset (*Pantherophis alleghaniensis/quadrivittatus*) from Burbrink et al. (2021).

Figure S6. (A) Ancestral species coefficients over geography, (B) probabilities of the number of clusters (K) and (C) data layer importance from climate-based SuperSOMs (alleles, space, and climate) in ‘delim-som’ (Pyron 2023) for the Kingsnake dataset (*Lampropeltis californiae/splendida*) from Myers et al. (2019).

Figure S7. (A) Ancestral species coefficients over geography, (B) probabilities of the number of clusters (K) and (C) data layer importance from climate-based SuperSOMs (alleles, space, and climate) in ‘delim-som’ (Pyron 2023) for the Diamondback Rattlesnake dataset (*Crotalus atrox*) from Schield et al. (2015).

Figure S8. Bivariate scatterplots showing the relationship between ancestry/admixture estimates from TESS3r (described above) versus the species coefficients from climate-based SuperSOMs (alleles, space, and climate) in ‘delim-som’ (Pyron 2023). For the Milk Snakes (*Lampropeltis triangulum/gentilis*) we overlaid estimates from the two different datasets of Burbrink et al. (2022) in black and Chambers et al. (2023) in gray.

Figure S9. Graphs showing estimated cline widths and cline centers for all loci between species-pair comparisons using HZAR.

Figure S10. A) The location of lineages and interpolated contour clines defining the extent of hybrid zones, B) loess plot showing individual distance to the cline center against admixture, C) cline estimates from HZAR, and D) density of admixture when combining admixture data from Burbrink et al. (2022) and Chambers et al. (2023) using TESS3r for *Lampropeltis triangulum* and *L. gentilis*.

Figure S11. The number of loci significantly correlated to changes in indicated environmental variables for each species pairs using redundancy analyses (RDA).

Figure S12. Maps showing the location of lineages using only loci that are significant among genome clines, genome scans, and DAPC for all lineage pairs. Values above each graph show the number of loci (and percentage of total loci used here) and Fst values for those reduced loci between geographic lineages.
